# A Reductionist Molecular Dynamics Framework for Identifying Molecular Descriptors of Hydration at Solid–Liquid Biomaterial Interfaces

**DOI:** 10.3390/polym18212677

**Published:** 2026-10-28

**Authors:** Tomoya Nakago, Kohei Shiraishi

**Affiliations:** Systems Engineering, Graduate School of Systems Engineering, Kindai University, 1 Umenobe, Takaya, Higashi-Hiroshima 739-2116, Hiroshima, Japan; 2544830001v@hiro.kindai.ac.jp

**Keywords:** molecular dynamics, biomaterial, biocompatibility, hydrogen bond, hydration, LAMMPS

## Abstract

In synthetic polymer-based biomaterials, interfacial hydration plays a crucial role in biological interactions with proteins and cells. Although a wide range of polymers, including zwitterionic and non-ionic types, are used as biomaterials, the molecular principles by which their chemical structures influence interfacial hydration and suppress nonspecific protein adsorption remain unclear, hindering the discovery of novel biomaterials. In this study, we developed a reductionist bulk solid–liquid molecular dynamics (MD) framework that deliberately excludes system-specific factors such as polymer stereoregularity. Non-ionic, amino-acid-based zwitterionic, betaine-based zwitterionic, and highly polar polymers were evaluated using established hydrogen-bond (HB) analyses. HB distance and angle distributions, HB number, radial distribution functions (RDF), HB lifetimes, and HB dissociation–reformation dynamics were analyzed to characterize the spatial and temporal behavior of interfacial hydration. These analyses revealed distinct hydration profiles among the polymer systems. The rk_f_/rk_b_ ratio was examined as a candidate descriptor characterizing the relative timescales of HB dissociation and reformation. Although polymer-dependent differences were observed under the present simulation conditions, their quantitative convergence and relationship with biological performance remain to be established. The ratio may therefore provide a useful starting point for future studies aimed at developing hydration-related molecular descriptors for AI/ML-assisted screening. Overall, the proposed reductionist framework provides a practical strategy for identifying hydration-related molecular descriptors using established MD analyses.

## 1. Introduction

Highly biocompatible surface modification strategies are essential for the safe and minimally invasive use of medical devices, where interfacial interactions between materials and biological components critically determine clinical performance. Among polymeric biomaterials, hydration-mediated suppression of nonspecific biological interactions is a well-established strategy for achieving high biocompatibility, with interfacial water structure considered an important factor governing biological performance. Zwitterionic betaine-based polymers are broadly classified into phosphobetaine, carboxybetaine, and sulfobetaine types [1,2,3,4,5,6,7]. Among these, phosphorylcholine-containing polymers, including poly(2-methacryloyloxyethyl phosphorylcholine) (PMPC) and MPC-based copolymers, have been extensively investigated because of their highly hydrated interfaces and their ability to suppress nonspecific biological interactions [8,9,10,11,12,13]. MPC-based copolymers have also been investigated for surface modification of blood-contacting and implantable medical devices [11,12,13]. In addition to conventional betaine-based polymers, our previously reported systems include amino-acid-based amphoteric polymers, such as poly(L-lysine (meth)acrylamide)s, which have demonstrated high biocompatibility associated with their unique interfacial charge distributions [14,15,16,17]. Representative nonionic polymers include poly(2-methoxyethyl acrylate) (PMEA), developed by Tanaka and co-workers [18], and poly(vinylpyrrolidone) (PVP), reported by Haaf and colleagues [19,20]. In addition to bound and free water, certain biocompatible polymers have been proposed to exhibit a distinct hydration state termed “intermediate water” [21,22,23]. Intermediate water is considered to form a relatively stable hydration layer that suppresses direct contact between biomolecules and the polymer surface. However, the molecular-level principles that connect specific interfacial hydration structures to macroscopic biocompatibility remain unresolved. Hydration structures have been characterized using techniques such as Fourier-transform infrared spectroscopy (FT-IR), Raman spectroscopy [24,25], nuclear magnetic resonance (NMR) and differential scanning calorimetry (DSC). These experimental approaches have provided important insights into hydration structure and dynamics; however, their measured observables are often material-specific or spatially averaged, making it challenging to define a common set of molecular descriptors that enables systematic comparison of hydration properties across chemically diverse polymer systems. Molecular dynamics (MD) simulations provide a means of characterizing polymer–water interactions at the molecular level and may therefore facilitate the identification of molecular descriptors associated with interfacial hydration. Previous computational studies, including molecular dynamics simulations and density functional theory calculations, have provided valuable molecular-level insights into polymer–water interactions [26,27,28,29,30]. However, such configurations do not directly reproduce the spatial confinement and interfacial organization characteristic of solid polymer surfaces used in coating-type biomedical materials. Consequently, systematic investigations specifically designed to represent coating-like solid–liquid interfaces remain relatively limited. This distinction is important because differences in interfacial hydration environments may influence biological responses at material interfaces. While hydration in liquid–liquid systems occurs in three dimensions around freely dispersed molecules, hydration at solid–liquid interfaces is confined to an interfacial region with distinct structural and dynamical properties. Such differences in interfacial hydration have been reported to affect protein adsorption, protein conformation, and subsequent cellular responses [31,32]. In our previous study, we demonstrated that the activity of fibrinolytic enzymes interacting with lysine-containing polymers differed between liquid-phase and solid-phase conditions, suggesting that interfacial hydration and molecular organization significantly influence protein function [15]. Furthermore, unlike polymer chains freely dispersed in solution, polymer chains constituting a solid material are spatially constrained by neighboring chains, resulting in different molecular mobility and interfacial organization. Consequently, the local hydration environment and the accessibility of functional groups at the interface are also expected to differ substantially from those in liquid–liquid systems. These differences indicate that solid–liquid models can provide a more directly relevant representation of the interfacial environment of coating-type biomaterials and therefore complement existing molecular models of polymer–water interactions. In this study, we developed a reductionist molecular dynamics framework for the systematic exploration of molecular descriptors associated with hydration at solid–liquid biomaterial interfaces. Rather than attempting to reproduce the full physicochemical complexity of experimentally prepared polymer interfaces, the model was intentionally simplified by standardizing or simplifying factors such as stereoregularity, atomic partial-charge distributions, water content, and swelling behavior. This reductionist strategy was designed to facilitate systematic comparison among chemically diverse polymers and to isolate molecular features associated with interfacial hydration. Solid–liquid models comprising water and nonionic, amino-acid-based, betaine-type, and highly polar polymers were therefore constructed within a common modeling framework. Candidate hydration descriptors were systematically evaluated from complementary structural and dynamic characteristics of polymer–water interactions, including radial distribution functions, hydrogen-bond populations, hydrogen-bond geometries, hydrogen-bond lifetimes, and hydrogen-bond formation and dissociation kinetics. Particular attention was given to the ratio of the characteristic times for HB dissociation and reformation (rk_f_/rk_b_) as a candidate descriptor of interfacial HB exchange dynamics. Through this systematic descriptor exploration, we aimed to identify molecular parameters that can distinguish hydration states across chemically diverse polymer interfaces and may be relevant to hydration-related aspects of biocompatibility. The resulting reductionist framework is not intended to reproduce every feature of a specific experimental material, but rather to provide a tractable platform for molecular descriptor screening that can complement detailed mechanistic studies of individual biomaterial systems and support future integration with AI/ML-assisted molecular discovery and the rational design of biocompatible polymeric materials.

## 2. Materials and Methods

### 2.1. Modeling and Calculations

**Scheme 1:**
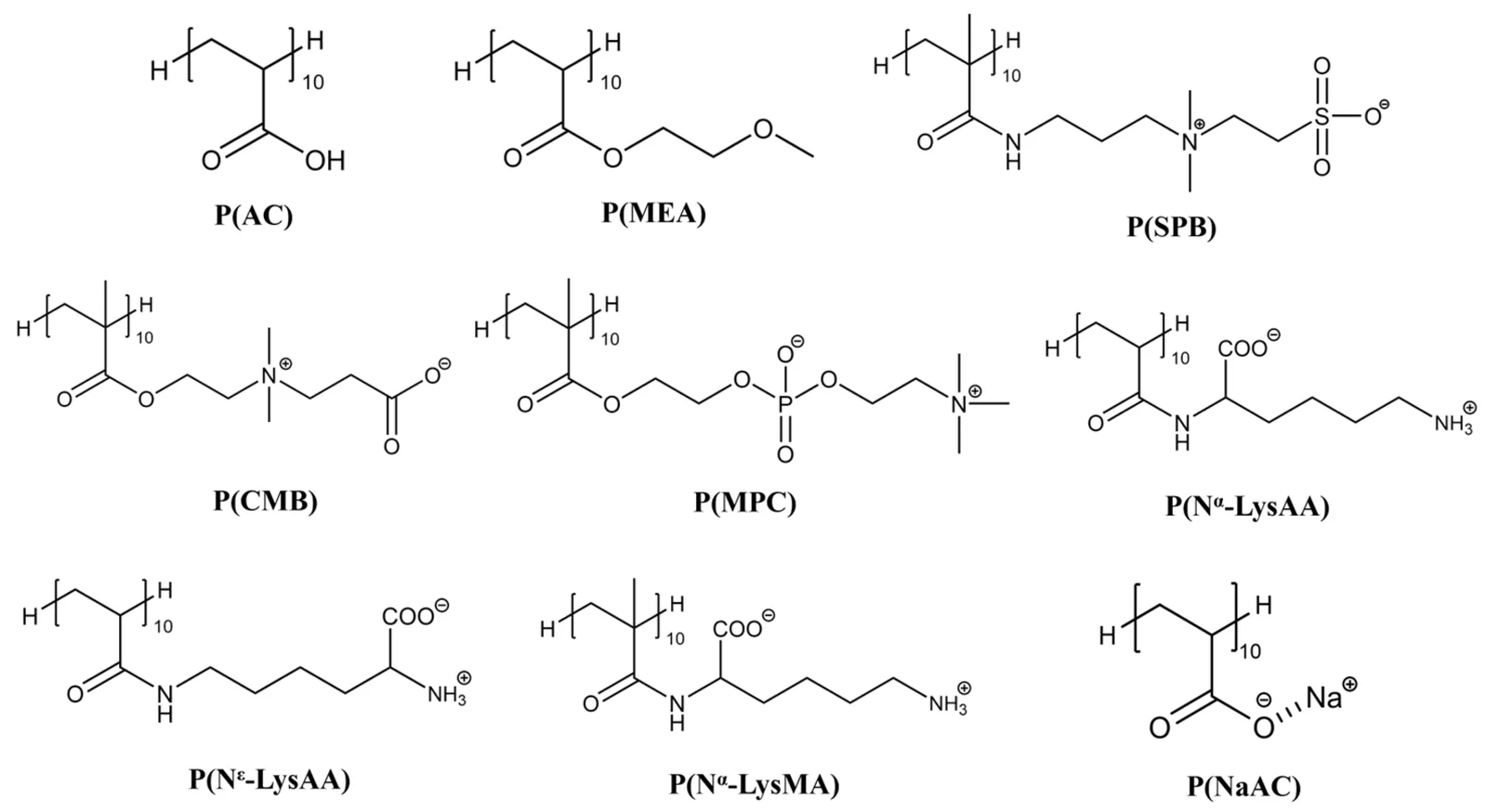
Chemical structures of the isotactic decamer models used in the molecular dynamics simulations.

To investigate the hydration structure and dynamics at solid–liquid polymer–water interfaces, molecular dynamics (MD) simulations were performed using the LAMMPS simulation package [33,34] on the “Genkai” supercomputer at Research Institute for Information Technology, Kyushu University. The following polymers were employed as representative models: poly(2-methoxyethyl acrylate) (P(MEA)) [18], poly(acrylic acid) (P(AC)), poly(N,N-dimethyl-N-(3-sulfopropyl)-3′-methacrylamidopropanaminium inner salt) (P(SPB)) [4,5], poly(1-carboxy-N,N-dimethyl-N-(2′-methacryloyloxyethyl)methanaminium inner salt) (P(CMB)) [2], poly(2-methacryloyloxyethyl phosphorylcholine) (P(MPC)) [1], poly(N^α^-L-lysine acrylamide) (P(N^α^-LysAA)) [14,15], poly(N^ε^-L-lysine acrylamide) (P(N^ε^-LysAA)) [15], poly(N^α^-L-lysine methacrylamide) (P(N^α^-LysMA)) [14,17], and poly(sodium acrylate) (P(NaAC)). These polymers were selected to represent nonionic, zwitterionic, amino-acid-based amphoteric, and highly polar polymer systems (Scheme 1). To standardize the models, P(AC) was represented in its protonated carboxylic acid form (–COOH), whereas P(NaAC) was represented using a simplified sodium carboxylate configuration containing COO^−^ and an associated Na^+^ group. The simulations did not explicitly account for pH-dependent ionization equilibria or counterion dissociation. Accordingly, the conclusions for these two systems are limited to the specific charge-state and topological representations employed here.

To facilitate a systematic comparison of their intrinsic hydration characteristics, all polymer models were constructed as isotactic decamers. This approach minimized the influence of stereoregularity-dependent variations in side-chain arrangement on the interfacial hydration behavior. Thirty polymer chains were initially packed into the simulation cell using randomized molecular placement with a minimum non-bonded interatomic distance of 0.15 nm. Partial atomic charges of the polymers were assigned using the AM1-BCC method. Polymer force fields were described using GAFF2. For P(NaAC), the Universal Force Field (UFF) was applied due to limitations in parameter availability. Each polymer system was treated independently using a single consistent force-field framework. Water molecules were modeled using the SPC/E water model. Approximately 3000 water molecules were added to each simulation cell to construct the hydrated interfacial systems. Energy minimization was first performed for each system, followed by equilibration under NVT and NPT ensembles at 310.15 K (37 °C) and 1 atm. Thermal annealing was omitted to maintain a common, simplified preparation protocol across polymer systems. Instead, the polymer phase was initially packed at a nominal density of 1.5 g cm^−3^, resulting in system-dependent cell dimensions. This standardization facilitated comparisons within the reductionist framework but did not ensure equilibrium packing structures or eliminate differences in polymer-specific density and morphology. Thermal-annealing procedures relevant to polymer equilibration have been reported previously [35]. Periodic boundary conditions were applied in all three spatial directions (x, y, and z). Nonbonded interactions were evaluated using the Lennard–Jones potential and Coulomb interactions (lj/cut/coul/long), with a cutoff distance of 1.2 nm for both van der Waals and short-range electrostatic interactions. Long-range electrostatic interactions were calculated using the particle–particle particle–mesh (PPPM) method with a relative force accuracy setting of 1 × 10^−6^ and a fourth-order interpolation scheme. No slab-specific electrostatic correction was applied. Arithmetic mixing rules and AMBER-type scaling of intramolecular nonbonded interactions were employed. After constructing the solid–liquid interfacial model, the systems were further equilibrated under the same thermodynamic conditions (310.15 K, 1 atm) to ensure stabilization of the interfacial structure (Figures S2 and S3). The stabilization of the interfacial density profiles along the surface-normal direction was monitored as an indicator of structural equilibration. Following equilibration, production runs were carried out under the NPT ensemble for 1 ns. The equations of motion were integrated with a timestep of 1 fs. During the NPT production simulations, temperature and pressure were controlled using Nosé–Hoover thermostat and barostat algorithms, respectively. The temperature and pressure were maintained at 310.15 K and 1 atm, with damping times of 100 fs for both controls. The thermostat and barostat chain lengths were set to three. Isotropic pressure coupling was applied along all three spatial directions. The SHAKE algorithm was employed for selected bond and angle constraints with a tolerance of 1 × 10^−6^ and a maximum of 1000 iterations. Trajectories were recorded at 1 ps intervals for analysis. The final simulation-cell dimensions obtained after the 1 ns NPT production runs are provided in Figure S1 of the Supplementary Information. Furthermore, the temporal stability of the polymer–water interface models was evaluated based on fluctuations in the HB numbers. The 1 ns NPT production trajectory was divided into five consecutive 200 ps blocks, and the mean HB population was calculated for each block. The block means were compared with the overall mean obtained from the entire 0–1000 ps trajectory, and their standard deviation (SD) and coefficient of variation (CV) were calculated to assess temporal variability. A CV of 3% was used as a practical target for temporal stability, with values below 5% considered acceptable for the present exploratory analysis. The block-averaged HB populations and the corresponding SDs, SEs, and CVs are provided in Figure S5, while statistics obtained from the full 0–1000 ps trajectories are summarized in Figure S6. Although all systems satisfied the adopted block-CV threshold, some exhibited systematic changes in HB populations across the trajectory. Thus, these results indicate limited temporal variability in HB populations under the present conditions but do not establish complete equilibration, convergence of HB correlation functions, or between-replicate reproducibility. Only one 1 ns production trajectory was evaluated for each polymer system. Detailed density profiles are provided in Figures S1–S4 in the Supplementary Information.

### 2.2. Hydrogen-Bond Analysis

Hydrogen-bond (HB) analyses were performed using GROMACS version 5.0.7 [36] through the Winmostar molecular modeling platform (X-Ability Co., Ltd., Tokyo, Japan).

#### 2.2.1. Hydrogen-Bond Number Analysis

The number of HBs was evaluated using the gmx hbond module implemented in GROMACS (version 5.0.7). An HB was defined using a donor–acceptor distance cutoff of 0.35 nm and a hydrogen–donor–acceptor deviation-angle cutoff of 30°. The gmx hbond -num option counts all donor–hydrogen–acceptor triplets satisfying these geometric criteria. Because each water molecule contains two hydrogen atoms, multiple donor–acceptor combinations are possible between a pair of water molecules. For example, two water molecules can form up to four possible D–H···A combinations depending on their relative orientation. To examine longer-range intermolecular correlations, an extended donor–acceptor distance cutoff of 1.0 nm was additionally evaluated. Interactions identified at distances beyond 0.35 nm were not interpreted as conventional hydrogen bonds, but rather as longer-range intermolecular correlations. Detailed HB analyses for individual components and additional cutoff conditions (e.g., 0.70 nm) are provided in the Supplementary Information (Figures S7 and S8).

#### 2.2.2. Hydrogen-Bond Distance Distribution

The distance distribution of HBs was evaluated using the gmx hbond -dist module in GROMACS (version 5.0.7). For this analysis, an angular cutoff of 30° (deviation angle) was applied, and donor–acceptor distances up to 1.0 nm were sampled to obtain the distribution profile. The donor–acceptor distance distributions were normalized by the total number of sampled configurations and the distance bin width (0.005 nm), yielding probability densities in units of nm^−1^. Interactions identified at distances beyond 0.35 nm were not interpreted as conventional HBs, but rather as long-range electrostatic/molecular interactions. Because the distributions were normalized histograms, peak intensities were interpreted qualitatively rather than as absolute HB populations. Radial distribution functions (RDFs) were calculated using the gmx rdf module in GROMACS (version 5.0.7) for each polymer relative to H_2_O. The RDFs were normalized by the number of reference atoms, the spherical-shell volume, and the average number density of target atoms, yielding dimensionless values. The distance bin width was 0.002 nm. Because the polymer–water systems were spatially inhomogeneous, these RDFs were interpreted as reference-dependent, normalized pair distributions rather than direct density profiles along the surface normal.

#### 2.2.3. Angular Distribution of Hydrogen Bonds

The angular distribution of HBs was evaluated using the gmx hbond -ang module in GROMACS (version 5.0.7). The donor–acceptor distance cutoff was fixed at 0.35 nm, and the full angular range (0–180°) was sampled for the hydrogen–donor–acceptor (H–D–A) deviation angle. Angular distributions were evaluated for both polymer–water and water–water HBs using the same geometric criteria (distance cutoff 0.35 nm). This allows direct comparison between interfacial and bulk-like HB geometries. Two angular conventions are commonly used in HB analysis: the absolute H–D–A angle and the deviation angle. In this study, the deviation angle representation was employed, where 0° corresponded to a perfectly linear HB (i.e., an H–D–A angle of 180°). Increasing deviation angles therefore indicate increasing distortion from linearity. In this representation, smaller deviation angles indicate more linear HBs. Because this analysis provides normalized histogram distributions, it enables qualitative comparison of angular preferences but does not provide an absolute quantitative measure based solely on peak intensities. The resulting histograms were normalized by the total number of sampled configurations and the angular bin width (1°), yielding angular probability densities in units of degree^−1^. The integrated angular population was calculated as

(1) $$I_{\theta 0.5-\theta 179.5}\approx \;\sum_{i=a}^{b-1}\left[\frac{P\left(\theta_{i}\right)+P\left(\theta_{i+1}\right)}{2}\right]\left(\theta_{i+1}-\theta_{i}\right)\;$$

where *P*(*θ_i_*) represents the normalized angular probability density at angle *θ_i_*. Numerical integration was performed over the entire angular range of 0.5–179.5°. Subsequently, the interval areas corresponding to the nearly linear angular peak (0.5–35.5°) were summed and used for correlation analysis with HB/Pairs and Degree of HB formation without further normalization. The resulting integrated angular population represents the relative contribution of nearly linear configurations to the overall angular distribution.

#### 2.2.4. Hydrogen-Bond Correlation and Kinetic Analysis

HB correlation and kinetic analyses were performed using the gmx hbond -ac module implemented in GROMACS 5.0.7, with a donor–acceptor distance cutoff of 0.35 nm and an angular deviation cutoff of 30°. Additional analyses using an extended distance cutoff of 1.0 nm are provided in the Supplementary Information (Figures S9–S11). The intermittent HB autocorrelation function and the HB–non-HB contact cross-correlation function were employed to characterize the temporal behavior of polymer–water interactions. The intermittent HB autocorrelation function, denoted by *A_c_*(*t*), is defined as

(2) $$A_{c}\left(t\right)=\;\frac{\langle h\left(0\right)h\left(t\right)\rangle }{\langle h\left(0\right)\rangle }$$

where *h*(*t*) is a binary HB existence function that takes a value of 1 when a donor–acceptor pair satisfies the geometric HB criteria and 0 otherwise. The angular brackets denote ensemble and time-origin averaging. This function describes the temporal correlation of HB configurations, including contributions from HBs that reform after dissociation [37,38,39].

In contrast, the HB–non-HB contact cross-correlation function, denoted by *C*_*contact*_(*t*), is expressed as

(3) $$C_{\mathrm{contact}}\left(t\right)=\;\frac{\langle h\left(0\right)\left[1-h\left(t\right)\right]H\left(t\right)\rangle }{\langle h\left(0\right)\rangle }$$

where *H*(*t*) is a binary contact function that takes a value of 1 when the donor–acceptor pair is within the specified contact distance and 0 otherwise. The term [1 − *h*(*t*)]*H*(*t*) therefore represents a non-HB contact state in which the donor–acceptor pair remains spatially proximate but does not satisfy the geometric criteria for HB formation. Accordingly, the contact cross-correlation function characterizes the temporal relationship between initially hydrogen-bonded configurations and subsequent non-HB contact states rather than the uninterrupted lifetime of individual HBs. The numerical normalization of the correlation functions followed the implementation in GROMACS (version 5.0.7). The HB dissociation and reformation rate constants, k_f_ and k_b_, respectively, were evaluated using the Luzar–Chandler kinetic model based on the intermittent HB autocorrelation and HB–non-HB contact cross-correlation functions [37,40]. Within this framework, the temporal evolution of HB correlations is related to the dissociation and reformation processes. The corresponding characteristic times were defined as the reciprocals of the rate constants: rk_f_ = 1/k_f_ and rk_b_ = 1/k_b_. The ratio rk_f_/rk_b_ was used as a relative index of the balance between HB dissociation and reformation timescales. In addition, the integrated HB correlation time, τ_HB_, was obtained from the time integral of the tail-corrected intermittent HB autocorrelation function. Unlike an uninterrupted HB lifetime, this correlation time reflects the overall relaxation behavior of HB correlations, including contributions from dissociation and subsequent reformation. Accordingly, τ_HB_ and rk_f_/rk_b_ were evaluated as complementary indices characterizing different aspects of HB dynamics. The finite-time integrals of the intermittent HB autocorrelation and HB–non-HB contact cross-correlation functions shown in Figure 4D and Figure 5D, respectively, were calculated using the trapezoidal rule, as employed in the angular distribution analysis (Section 2.2.3). These integrated values were used for comparative analysis and were not interpreted as uninterrupted HB lifetimes. The intermittent HB autocorrelation functions exhibited incomplete decay within the simulation time window for several polymer systems, indicating residual temporal correlations at the polymer–water interface. However, these residual correlations raise uncertainties regarding the quantitative convergence of the kinetic parameters obtained from the Luzar–Chandler model. Furthermore, the long-time behavior of the contact cross-correlation functions may contain contributions from non-HB contact-state populations and should not be interpreted as uninterrupted HB persistence. Consequently, the resulting kinetic parameters and the rk_f_/rk_b_ ratio were interpreted as relative measures of HB relaxation and exchange dynamics rather than fully converged absolute kinetic quantities. Previous NMR studies have reported substantial differences in molecular rotational correlation times τ_c_ among free (0.001–0.01 ns), intermediate (0.1–1 ns), and strongly bound water (10–1000 ns), with the latter exhibiting considerably slower dynamics [41]. These observations suggest that complete sampling of slowly relaxing hydration components may require simulation times substantially longer than those employed in the present study. Accordingly, the 1 ns production simulations were intended to provide a computationally tractable framework for exploring relatively fast interfacial HB dynamics rather than fully resolving the long-time relaxation behavior of strongly bound water. The resulting kinetic parameters should therefore be interpreted as candidate descriptors evaluated within the present sampling conditions, and their quantitative convergence requires further investigation.

## 3. Results and Discussion

**Figure 1:**
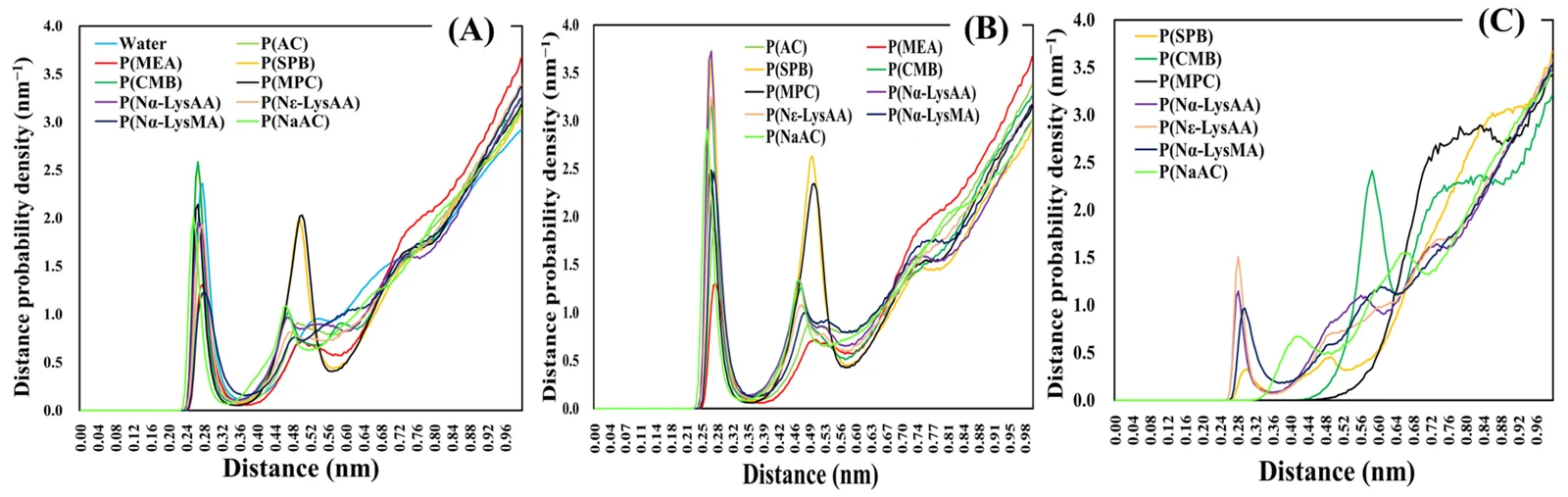
Hydrogen-bond distance distribution analysis for each polymer (up to ~1.0 nm). (**A**) HB distance distribution for all atoms. (**B**) HB distance distribution of the anionic components in the polymers (O or OH). (**C**) HB distance distribution of the cationic components in the polymers (N or NH). Polymers lacking cationic components were excluded from panel (**C**).

Figure 1 shows the HB distance distribution analysis relative to the polymer–water interface, evaluated within a 30° angular cutoff, over the range of 0–1.0 nm. We considered that this analysis enables a qualitative evaluation of the spatial organization of interfacial water associated with hydrogen bonding and electrostatic interactions. Based on the peaks in Figure 1, the interfacial hydration region within 1.0 nm can be divided into four layers: Layer 1 (0.24–0.35 nm), Layer 2 (0.40–0.55 nm), Layer 3 (0.60–0.85 nm), and Layer 4 (>0.90 nm). The results indicate that both the characteristic HB distances and the distribution profiles depend on the type and polarity of functional groups, such as oxygen and nitrogen, present in the polymer. Polymers with highly polar groups, including P(MPC) and P(SPB) containing phosphorylcholine and sulfonate groups, exhibited pronounced peaks in both the first and second hydration layers, consistent with experimental observations of substantial non-freezing and intermediate water associated with phosphorylcholine-containing polymers [42]. For betaine-type and cationic polymers, the characteristic hydration peaks were shifted toward shorter distances from the polymer interface relative to the nonionic and amino-acid polymers in the first hydration layer. In contrast, polymers possessing less strongly polar oxygen-containing groups exhibited weaker HB-associated distance distributions in the second hydration layer. Four polymers, consisting of one nonionic polymer, P(MEA), and three amino-acid-based zwitterionic polymers, exhibited HB distance distributions in their hydration layers that were similar to that of bulk water, in contrast to the betaine-type polymers. Recent experimental measurements using Overhauser dynamic nuclear polarization have likewise demonstrated that water near zwitterionic polymer surfaces can display relatively fast, bulk-like translational dynamics, depending on the zwitterion chemistry and ionic environment [43]. As distinct peaks were observed up to the second hydration layer, these results suggest the formation of a more clearly structured hydration environment. Although these geometrically defined layers cannot be directly equated with experimentally defined bound, non-freezing, or intermediate water, they provide a molecular-scale spatial framework for comparing hydration structures across polymer systems. For comparison, previous studies have similarly shown that interfacial water ordering can extend several angstroms from strongly interacting surfaces [44]. In addition, experimental studies using atomic force microscopy (AFM) have shown that hydration layers at solid–liquid interfaces can form two to four layers separated by approximately 0.3–0.5 nm, depending on the material properties [31]. Although the peak intensities varied with the polymer functional groups, three to four characteristic peak regions were observed at broadly similar distances across the investigated polymers. These regions were classified sequentially using common distance ranges to facilitate structural comparisons, consistent with the concept of multilayer interfacial hydration reported in previous AFM studies [31]. However, the donor–acceptor distance distributions do not directly represent spatial hydration layers. Furthermore, this classification was used only for interpreting distance distributions and was not applied to the subsequent HB population, angular distribution, or kinetic analyses. Therefore, the principal quantitative descriptors are not affected by the selected boundaries between the four regions. These observations suggest that the mechanisms governing interfacial hydration may differ among betaine-type, amino-acid-based zwitterionic, and nonionic polymers, depending on the polarity and charge characteristics of their functional groups. Analysis of the HB distance distributions revealed distinct spatial characteristics for the anionic and cationic groups within the polymer molecules. Anionic groups exhibited HB-associated features at relatively short distances, with characteristic peaks appearing from approximately 0.2 nm. In contrast, cationic groups exhibited broader hydration features extending to longer distances than those observed for anionic groups. This difference is consistent with trends reported in radial distribution function (RDF) analyses of anion–cation interactions involving poly(ethylene oxide) in an imidazolium-based ionic liquid, as reported by Dan et al. [45]. The more distant interactions of the non-protonic cationic groups are likely due to steric hindrance from adjacent methyl substituents. Q. Shao et al. reported the spatial distribution of water molecules around carboxybetaine and sulfobetaine, showing that methyl substituents can restrict the approach of water molecules to cationic groups from certain directions [46]. Water molecules are strongly polarized toward oxygen, which facilitates extended hydration with the cationic groups of the polymer. In contrast, protonated cationic groups such as –NH_3_^+^ do not possess the same steric environment as quaternary ammonium groups and may therefore permit closer approach of water molecules. Furthermore, Drici reported from RDF profiles that the characteristic hydration distances of amino-acid-specific carboxylate (–COO^−^) and ammonium (–NH_3_^+^) groups in the liquid phase remained relatively similar. They also demonstrated that the hydration distance varies depending on the structure of the amino acid side chain [47]. In the present study, Figure 1B,C show that the anionic (–COO-) and cationic (e.g., –NH3^+^) groups of amino-acid-based polymers exhibited similar characteristic peaks extending to the second hydration layer, consistent with the trends reported in previous studies.

**Figure 2:**
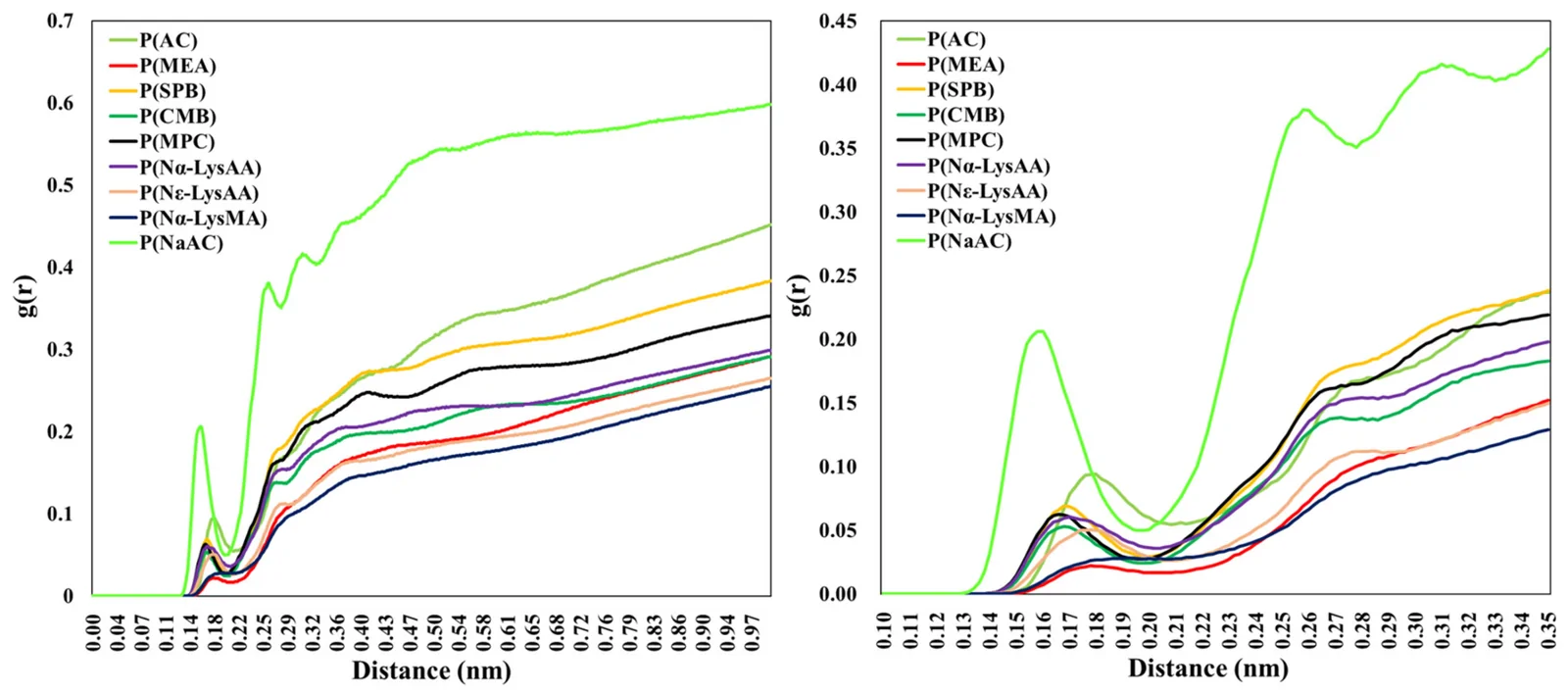
Radial distribution function (RDF) profiles of each polymer. (**Left**) RDF profiles up to 1.0 nm from the polymer–water interface. (**Right**) Enlarged view of the interfacial region up to 0.35 nm.

Figure 2 shows the radial distribution function (RDF) profiles for each polymer. To facilitate direct comparison with the HB distance distributions shown in Figure 1, the RDF analysis was limited to a distance of 1.0 nm from the polymer–water interface. The largest differences among the polymers examined within approximately 0.35 nm of the interface, corresponding to the first hydration layer. P(NaAC), a non-biocompatible, nonionic, and highly polar polymer, exhibited the highest and sharpest RDF peaks among all polymers. Four distinct peaks were identified, indicating pronounced spatial localization of water molecules at specific distances from the polymer interface. In contrast, the biocompatible betaine-type and nonionic polymers exhibited broader and less pronounced RDF peaks, except within the first hydration layer at 0.14–0.20 nm. These broader peaks indicate a less localized spatial distribution of interfacial water molecules compared with P(NaAC), suggesting a less spatially confined hydration structure. Taken together with the HB distance distributions shown in Figure 1, these results suggest that biocompatible polymers form hydration structures that differ from those of highly polar polymers such as P(NaAC). While P(NaAC) forms a highly ordered and spatially localized hydration structure, the hydration layers of biocompatible polymers appear to retain local hydrogen-bonding interactions while exhibiting a more spatially diffuse distribution of interfacial water. These differences in the spatial organization of interfacial water may contribute to differences in hydration-related interfacial properties among the polymer systems.

**Figure 3:**
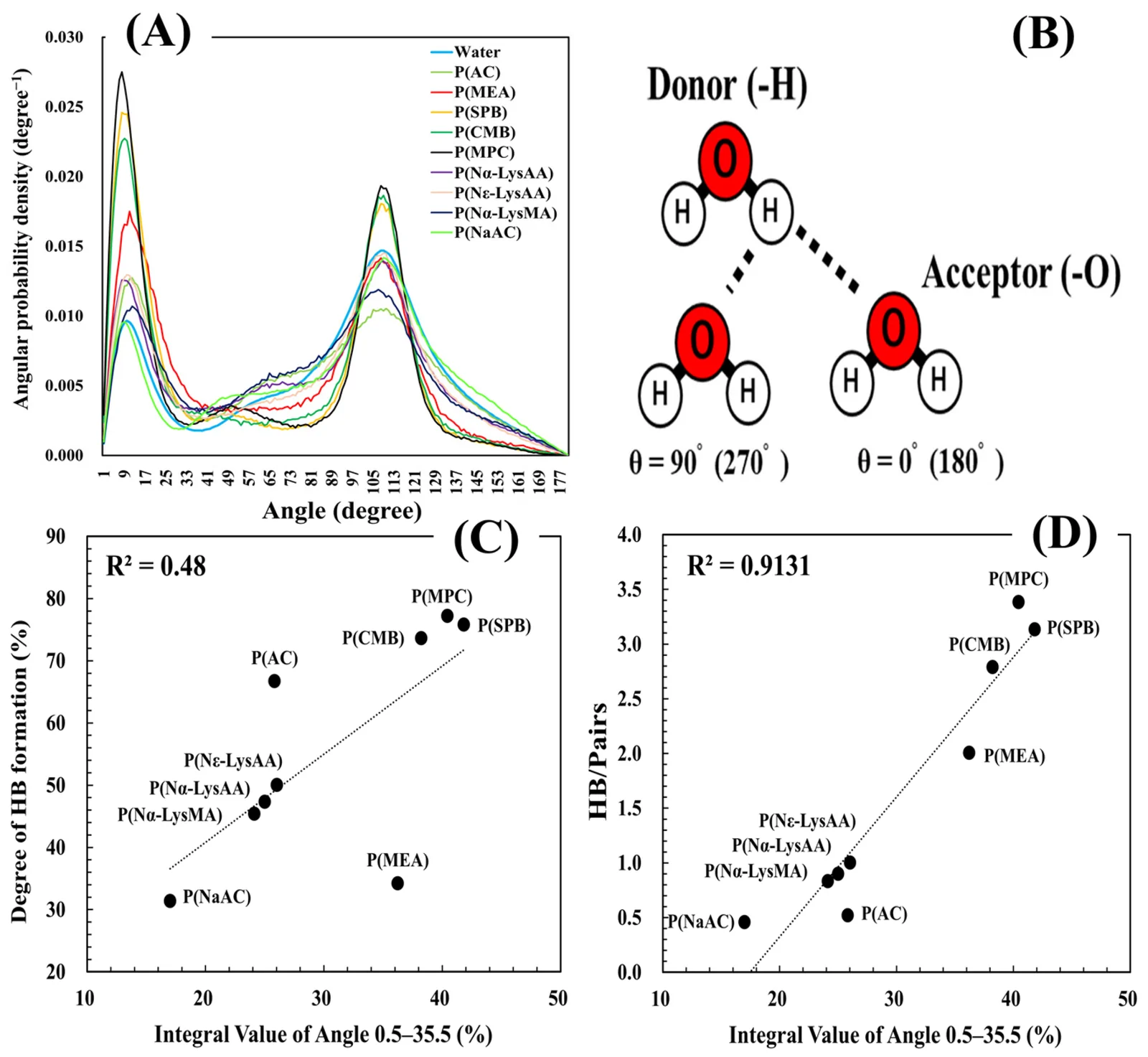
Angular analysis of hydrogen-bond geometries at the polymer–water interface. (**A**) Normalized angular distributions of hydrogen bonds for each polymer within 0.35 nm of the polymer–water interface. (**B**) Schematic representation of the hydrogen-bond geometry, where θ denotes the deviation angle (θ = 0° corresponds to a linear D–H···A configuration). (**C**) Correlation between the integrated population within the near-linear angular range (0.5–35.5°) and the “Degree of HB formation (%)” values reported in Table 2. (**D**) Correlation between the integrated population within the near-linear angular range (0.5–35.5°) and the “HB/Pairs” reported in Table 2.

Figure 3 presents the angular distribution of hydrogen bonds within 0.35 nm of the polymer–water interface, as obtained from the MD simulations. Compared with bulk water, the interfacial hydration structure exhibits a distinct angular distribution, consistent with the distance-based trends shown in Figure 1. Small deviation angles (≤35°) correspond to nearly linear HB geometries and are associated with more favorable hydrogen-bond configurations [48]. In contrast, deviation angles in the ranges of 36.5–89.5° and 131.5–179.5° represent increasingly distorted geometries. Interestingly, the intermediate angular region (90.5–130.5°) exhibits an enhanced population relative to the highly distorted ranges. This feature may reflect contributions from non-linear hydrogen-bond configurations within the interfacial water network. The observed angular characteristics can be considered in the broader context of the tetrahedral hydrogen-bond network of liquid water [49]. To further evaluate the structural relevance of the angular distributions, the area under the angular distribution curve within each defined angular range (Table 1) was calculated and compared with the “HB/Pairs” reported in Table 2. The integrated population within the near-linear angular region (≤35.5°) correlated with HB/Pairs (R^2^ = 0.9131). Because both quantities were calculated using related donor–acceptor geometries and the same underlying trajectories, this correlation should be interpreted primarily as geometrical consistency rather than independent validation of a molecular mechanism. In contrast, the correlation between the HB formation ratio and the distribution within the defined HB condition and angular regions was weaker (R^2^ = 0.480). When classified according to polymer type, amino-acid-based zwitterionic, betaine-type, nonionic, and highly polar non-ionized polymers occupied distinct regions, indicating that the relationship between HB formation and angular distribution differs among these classes. Notably, the six zwitterionic polymers exhibited a good linear correlation between the integrated population within the defined angular range and the degree of HB formation, suggesting that these polymers exhibit similar relationships between HB geometry and HB population. It should be noted that the highly polar polymers investigated in this study, P(AC) and P(NaAC), were intentionally modeled in simplified non-ionized states, although these polymers can undergo ionization under aqueous conditions. In particular, the biological response to P(AC) has been reported to depend on both polymer concentration and the pH of the surrounding medium, with changes in molecular weight also affecting the cellular response [50]. Neglecting ionization and its associated charge-state effects may therefore alter the hydrogen-bonding behavior relative to that expected under experimental aqueous conditions. Accordingly, the deviations observed for P(AC) and P(NaAC), including their weaker correlations, should be interpreted in the context of this modeling simplification.

Table 2 and Table 3 summarize the HB analysis performed under two distance criteria: 0.35 nm (classical HB cutoff) and 1.0 nm (long-range electrostatic/molecular interaction), both with a deviation-angle cutoff of 30°. The HB/Pairs values decrease with increasing distance from the polymer interface, indicating that linear HB-associated water populations are concentrated near the polymer surface. HB interactions are spatially localized near the polymer interface. Polymers reported to exhibit high biocompatibility tend to display highly ordered and preferentially oriented interfacial water molecules with enhanced HB stability. Highly ordered and preferentially oriented interfacial water may contribute to the formation of hydration states associated with biocompatibility. Such structured hydration layers may function as a dynamic water barrier during exchange processes with approaching proteins, thereby reducing direct polymer–protein contact. This interfacial water structuring could contribute to the suppression of nonspecific adsorption and subsequent protein denaturation. Kitano et al. used polarized Raman spectroscopy to analyze changes in the HB network of water in the presence of amphiphilic polymers and quantified the disorder in this network using the N^corr^ parameter [24,25]. There are two main factors that may explain the discrepancy between the simulation results of this study and the experimental N^corr^ values. The first factor is the difference in the systems and spatial environments examined by Raman spectroscopy and molecular dynamics (MD) simulations. While the MD model in this study characterizes the local hydration structure at the solid–liquid polymer interface, the Raman spectroscopy measurements reflect the collective hydrogen-bonding environment of water in an aqueous polymer solution. Consequently, differences in the spatial organization of water and the sampling environment may be one cause of the observed discrepancy. The second factor is the simplified treatment of the polymer structure and ionization state. In an aqueous environment, the degree of ionization of carboxyl groups depends on factors such as pH and ionic strength, and may affect electrostatic interactions at the interface, the hydration of counterions, and the organization of the hydrogen-bonding network. In this simulation, P(AC) was represented in its protonated form (-COOH), whereas P(NaAC) was modeled using a negatively charged carboxylate group (COO^–^-Na^+^) bound to a sodium ion. However, this was a simplified charge-state model without explicit counterion dissociation in which the effects of pH-dependent ionization equilibria and counterion dissociation were intentionally disregarded. Furthermore, all polymer chains were modeled as isotactic decamers, and the effects of variations in stereoregularity on the hydration structure and dynamics were excluded. Therefore, a direct quantitative agreement between the simulation-derived hydration descriptors and the experimental N^corr^ values cannot be expected. These simplifications may affect the predicted interfacial hydration structure and dynamics and could be a contributing factor to the discrepancies observed in experimental comparisons. Importantly, the results of this study do not directly represent hydration behavior or biocompatibility under physiological conditions; rather, they should be interpreted as characteristics of a reductionist model aimed at identifying candidate molecular descriptors. Incorporating experimentally relevant factors—such as ionization states, counterion distribution, and stereoregularity, including atactic chain arrangements—will be crucial for applying this framework to experimental biocompatibility studies or biomaterial systems. Independently, our analysis shows that the orientational ordering of HB water decreases sharply beyond ~0.70 nm from the polymer interface (Figures S7 and S8), corresponding to the second peak in the HB distance distribution shown in Figure 1. Within approximately 0.35 nm of the polymer interface, water molecules satisfying the conventional HB criteria were considered to constitute an HB-associated bound-water region in the present MD framework. The short donor–acceptor distances and near-linear HB geometries observed in this region indicate a geometrically favorable and strongly associated hydration environment. Beyond this region, the HB-associated water population gradually decreased, with the hydration structure becoming increasingly bulk-like at distances beyond approximately 0.70 nm. Water located between approximately 0.36 and 0.70 nm therefore represents a spatially distinct intermediate hydration regime between the strongly polymer-associated first hydration layer and the bulk-water-like outer region. These findings suggest that the formation of a weakly perturbed, intermediate-water-like hydration regime is associated with strong HB interactions near the polar polymer interface, primarily within the first one or two hydration layers, while the more distant hydration region remains comparatively bulk-like. From a molecular perspective, these results suggest that the hydration states observed experimentally at polymer interfaces may originate from the spatial organization and persistence of polymer–water interactions at the molecular scale. In this context, the present MD analysis provides structural descriptors of the molecular processes underlying interfacial hydration, rather than directly assigning experimentally defined hydration states. Thus, spatially confined HB-associated hydration, characterized by strong near-surface interactions and a gradual transition toward bulk-like water, may serve as a candidate molecular descriptor for comparing hydration environments among chemically diverse polymer surfaces and for relating molecular-scale hydration structure to hydration-related biocompatibility.

**Figure 4:**
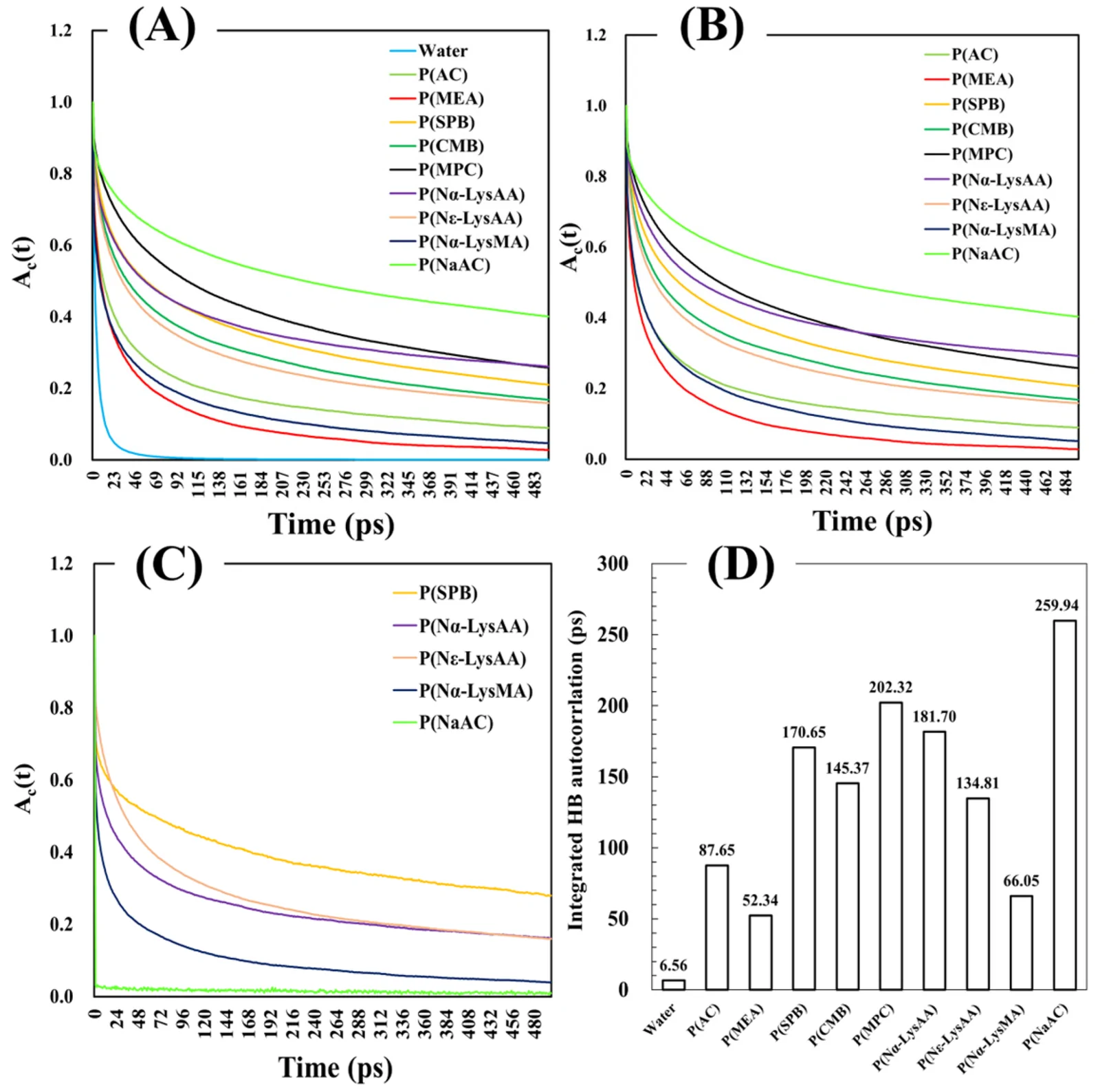
Intermittent hydrogen-bond autocorrelation analysis of polymer–water interactions. Cutoff conditions: Donor–acceptor distance = 0.35 nm; angular deviation for HB formation = 30°. (**A**) Intermittent HB autocorrelation functions for the entire polymer system. (**B**) Intermittent HB autocorrelation functions associated with the anionic components. (**C**) Intermittent HB autocorrelation functions associated with the cationic components. Systems exhibiting no detectable correlations under these conditions were excluded from the analysis. (**D**) Finite-time integrated values of the intermittent HB autocorrelation functions shown in panel (**A**), calculated using the trapezoidal rule, as employed in the angular distribution analysis (Section 2.2.3).

Figure 4 shows the HB lifetime profiles derived from the intermittent correlation function at the polymer–water interface, while Table 4 summarizes the corresponding rate constants k_f_ and k_b_ and their reciprocal characteristic timescales rk_f_ and rk_b_. Because the intermittent correlation function accounts for HB breaking and subsequent reformation, it provides information on the dynamic kinetics of HB exchange. In bulk water, the correlation function decays toward zero within the simulation time window. In contrast, the polymer models containing highly polar groups exhibited incomplete decay, indicating persistent HB correlations at the polymer–water interface. This incomplete decay shows that interfacial HB interactions persist for longer times than those in bulk water. Therefore, the Luzar–Chandler [37] kinetic model may not provide reliable absolute rate constants for the present systems, and the resulting kinetic parameters should be interpreted primarily as relative measures of HB exchange dynamics. The HB lifetime depended primarily on the accessibility and chemical nature of the polar side-chain groups. However, differences in the polymer backbone also influenced the HB lifetime, particularly among polymers possessing similar side-chain chemistries. Among the polymers reported to exhibit high biocompatibility, P(MPC) exhibited the longest HB lifetime (τ_HB_). The relatively long HB correlation time of P(MPC) may be associated with the accessibility of its terminal trimethylammonium group, which could contribute to persistent interactions with interfacial water compared with other betaine-type polymers. Among the amino-acid-based polymers examined in this study, P(N^α^-LysAA) showed the longest HB lifetime. It has been reported that, despite their non-specific plasma protein-binding properties, P(N^α^-LysAA) and P(N^α^-LysMA) can selectively interact with proteins involved in thrombolysis, such as plasminogen (Plg) and tissue-type plasminogen activator (tPA) [14,15,16,17]. Differences in interfacial water dynamics, including the rk_f_/rk_b_ ratio, between these polymers and other betaine polymers may reflect characteristic hydration dynamics associated with their selective interactions with these proteins. The structural similarity of P(N^α^-LysAA) to naturally occurring peptide structures may contribute to its favorable interfacial hydration behavior. In contrast, P(N^α^-LysMA), which possesses a methacrylate-based backbone, exhibits reduced backbone flexibility compared with P(N^α^-LysAA). This reduced conformational flexibility may limit persistent interfacial interactions with water and facilitate more rapid water reorientation and exchange, resulting in a shorter HB lifetime. As shown in Table 4, P(NaAC), represented by a simplified sodium carboxylate model, exhibited a relatively long integrated HB correlation time τ_HB_. The local electrostatic environment associated with the carboxylate groups and sodium ions may contribute to the stabilization of polymer–water interactions, resulting in prolonged characteristic times for HB dissociation rk_f_ and reformation rk_b_, as well as a relatively long τ_HB_. This behavior may be associated with the pronounced perturbation of the water HB network indicated by the relatively high N^corr^ value reported by Kitano et al. [24,25] (Table 2 and Table 3). However, because the experimental Raman measurements and the present MD simulations characterize different hydration environments, this correspondence should be interpreted qualitatively. The strong local electrostatic interactions in the simplified P(NaAC) model may influence the spatial organization and relaxation dynamics of interfacial water molecules, potentially contributing to the prolonged HB correlations observed in this system. Nevertheless, P(MPC) exhibited a comparable τ_HB_ value to P(NaAC), despite their different reported biocompatibility characteristics. Furthermore, the incomplete decay of the HB autocorrelation functions within the simulation time window introduces uncertainty into the calculated integrated correlation times and kinetic parameters. In particular, τ_HB_, obtained by integrating the tail-corrected intermittent HB autocorrelation function, may be sensitive to the treatment of residual long-time correlations. However, the derived rate constants k_f_ and k_b_ are also subject to sampling limitations. Therefore, the present τ_HB_, rk_f_, and rk_b_ values should be regarded as exploratory measures of interfacial HB dynamics rather than quantitatively converged lifetime or kinetic parameters. Interestingly, the τ_HB_ values of P(MPC), which exhibits excellent biocompatibility, and P(NaAC), for which no biocompatibility has been reported, were nearly identical. A similar trend was also observed for P(N_α_-LysMA), P(MEA) and P(AC). These observations suggest that τ_HB_, although an informative descriptor of interfacial HB dynamics, is not sufficient on its own to account for differences in biocompatibility among the polymer systems examined in this study. The HB lifetime associated with charged moieties varied depending on the type and spatial arrangement of the polar groups within the polymer. Bulky polar groups such as phosphate (–PO_4_^−^), sulfate (–SO_4_^−^), and carboxylate (–COO-), as well as cationic groups (secondary and primary amine), which are structurally accessible to interfacial water molecules, appear to promote stable HBs at the interface. Taken together, these results suggest that the chemical structure and accessibility of polar side-chain groups play important roles in modulating interfacial water structure and dynamics, which may in turn contribute to hydration-related aspects of biocompatibility. An intermittent correlation function calculated with an extended hydrogen-bond cutoff distance of 1.0 nm is presented in the Supplementary Information (Figure S9). Compared with the 0.35 nm condition, all systems exhibited a more gradual decay of the correlation function under the extended cutoff. This slower decay may indicate that intermolecular correlations beyond the conventional HB criterion contribute to the observed correlation function. In particular, systems dominated by long-range electrostatic effects indicate that non-protonic cationic groups may participate in stabilizing interfacial water, potentially leading to the formation of a thicker bound-water layer. To further characterize the exchange dynamics of interfacial water molecules, we analyzed the ratio rk_f_ /rk_b_. These rate constants are defined within the framework of the Luzar–Chandler model, in which HB (A) and non-HB (B) states are interconverted according to Equation (4) [40]. Here, k_f_ is the forward rate constant for the transition from the HB state (A) to the non-HB state (B), whereas k_b_ is the backward rate constant for the transition from B to A. Thus, k_f_ and k_b_ represent the rate constants for HB dissociation and reformation, respectively (Table 4 and Table 5).

(4) $$A\overset{k_{f}}{\underset{k_{b}}{⇄}}B$$

Because the rate constants k_f_ and k_b_ have units of ps^−1^, their reciprocals, rk_f_ and rk_b_, represent the characteristic timescales of HB dissociation and reformation, respectively. The ratio rk_f_/rk_b_ provides a measure of the dynamic equilibrium between HB dissociation and reformation at the polymer–water interface. The biocompatible polymers exhibited rk_f_/rk_b_ values in the range of 0.69–3.03. Therefore, a larger rk_f_/rk_b_ value indicates that dissociation occurs more slowly relative to reformation, corresponding to a reformation-dominant environment. When rk_f_/rk_b_ < 1, HB dissociation occurs on a shorter timescale than reformation, whereas rk_f_/rk_b_ > 1 indicates a relative preference for HB reformation. These results suggest that highly biocompatible polymers may be associated with a relatively balanced HB dissociation–reformation process or with a balance shifted toward HB reformation. In addition, among the polymers reported to exhibit high biocompatibility, those with rk_f_/rk_b_ ratios below unity may be more sensitive to changes in stereoregularity. Preliminary calculations using a model P(MPC) system indicated that stereoregularity may influence the rk_f_/rk_b_ ratio. However, a detailed investigation of this effect is beyond the scope of the present study. These observations suggest that the slight preference for HB dissociation observed in some biocompatible polymers may arise, at least in part, from differences in the accessibility of interfacial water associated with polymer stereoregularity. A previous study reported that polymer stereoregularity also influenced HB relaxation time [51]. Although P(MPC) and P(NaAC) exhibited nearly identical HB lifetimes in the present simulations, this similarity may be influenced by the simplified treatment of ionization states and atomic partial charges. Furthermore, Table 5 summarizes the HB lifetime parameters obtained from the analysis of water–water interactions in the water region of the polymer–water interface models, complementing the polymer–water HB analysis presented in Table 4. Because this analysis includes water molecules near the polymer interface, the resulting parameters reflect the influence of the polymer on water–water HB dynamics. By comparing these parameters with those obtained from a reference system containing only bulk water, we sought to establish a qualitative connection with the Raman spectroscopic observations reported by Kitano et al. [24,25]. The results revealed polymer-dependent changes in the rk_f_/rk_b_ ratio for water–water HBs. To characterize these changes, we introduced the Hydration Disruption Value (H_D_ value), defined by normalizing the rk_f_/rk_b_ ratio obtained for each polymer-containing system to that of reference bulk water. An H_D_ value close to unity indicates that the relative balance between HB dissociation and reformation timescales remains similar to that of reference bulk water. In contrast, an H_D_ value below unity indicates a shift toward relatively shorter HB dissociation timescales, whereas a value above unity indicates a shift toward relatively shorter HB reformation timescales. These deviations provide a comparative measure of polymer-induced changes in water–water HB dynamics, although they do not directly quantify the disruption of the HB network. Qualitative agreement was observed between the trends in H_D_ values for P(MPC), P(CMB), and P(NaAC) and the hydrogen-bond network disruption parameter N^corr^ reported from Raman spectroscopy. In contrast, P(AC) and P(SPB) did not follow the same trend. These discrepancies may partly arise from differences in ionization states, atomic partial-charge assignments, and polymer stereoregularity, which were simplified. Although the present reductionist framework has limitations in reproducing experimental polymer systems, these findings suggest that the H_D_ value may serve as a candidate molecular descriptor for comparing polymer-induced changes in water–water HB dynamics.

**Figure 5:**
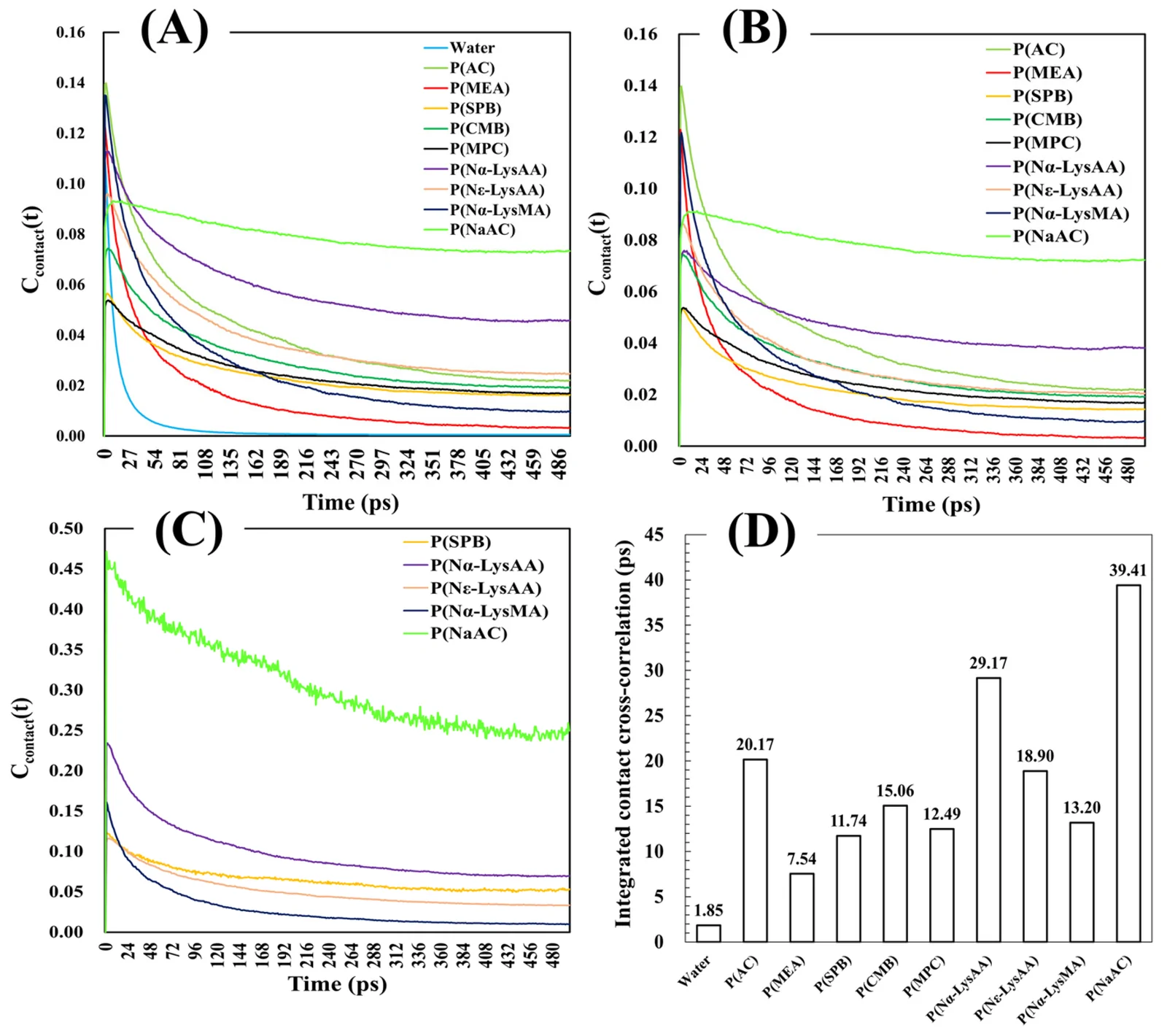
HB–non-HB contact cross-correlation analysis of polymer–water interactions. Cutoff conditions: Donor–acceptor distance = 0.35 nm; angular deviation for HB formation = 30°. (**A**) Contact cross-correlation functions for the entire polymer system. (**B**) Contact cross-correlation functions associated with the anionic components. (**C**) Contact cross-correlation functions associated with the cationic components. Systems exhibiting no detectable correlations under these conditions were excluded from the analysis. (**D**) Finite-time integrals of the contact cross-correlation functions shown in panel (**A**), calculated using the trapezoidal rule, as employed in the angular distribution analysis (Section 2.2.3).

Figure 5 shows the HB–non-HB contact cross-correlation functions at the polymer–water interface. These functions characterize the temporal correlation between initially hydrogen-bonded donor–acceptor pairs and their subsequent non-HB contact states, in which the pairs remain within the specified contact distance but no longer satisfy the geometric criteria for HB formation. As shown in Figure 5, the contact cross-correlation functions exhibited polymer-dependent temporal behavior, with appreciable long-time correlations remaining in several systems within the simulation time window. These residual correlations may reflect persistent associations between initially hydrogen-bonded configurations and subsequent non-HB contact states in the highly polar polymer environments. Because the correlation functions did not exhibit satisfactory long-time convergence, their quantitative interpretation remains limited. In particular, the finite-time integrals of the contact cross-correlation functions should not be interpreted as absolute HB lifetimes. P(NaAC) exhibited pronounced long-time correlations in both Figure 4A and Figure 5A, suggesting persistent correlations associated with both HB configurations and non-HB contact states at the polymer–water interface. This behavior may be related to the local electrostatic environment of the simplified sodium carboxylate model, which could favor the proximity of donor–acceptor pairs even when the geometric criteria for HB formation are no longer satisfied.

In contrast, the other polymer systems generally exhibited lower long-time contact cross-correlations, although differences remained in their initial decay behavior. In particular, the betaine-type polymers exhibited relatively low contact cross-correlations compared with P(NaAC), despite retaining appreciable intermittent HB autocorrelations in Figure 4. This difference suggests that the temporal relationships between HB configurations and non-HB contact states depend on the chemical characteristics of the polymer functional groups. The relatively low contact cross-correlations of the betaine-type polymers may reflect either the persistence or reformation of HB configurations or the separation of donor–acceptor pairs following HB dissociation. Therefore, these observations suggest differences in the dynamic rearrangement of polymer–water interactions, although they do not directly establish differences in water residence times or translational mobility.

Figures S9 and S10 show the intermittent HB autocorrelation and HB–non-HB contact cross-correlation functions, respectively, calculated using an extended donor–acceptor distance cutoff of 1.0 nm. Compared with the 0.35 nm criterion, the extended cutoff changed the time dependence of the calculated correlations, partly because additional geometrical configurations were included. These results should therefore be interpreted as cutoff-dependent contact and HB-correlation measures, rather than direct evidence for faster or slower water exchange. A comparison of rk_f_/rk_b_ and τ_HB_ reveals that the relative balance between HB dissociation and reformation does not necessarily follow the same trend as the integrated HB correlation time. This difference arises because k_f_ and k_b_ are obtained by applying the Luzar–Chandler kinetic model to the intermittent HB autocorrelation and HB–non-HB contact cross-correlation functions, whereas τ_HB_ is obtained from the time integral of the tail-corrected intermittent HB autocorrelation function [36]. Thus, τ_HB_ characterizes the overall temporal relaxation of HB correlations but does not separately quantify the contributions of HB dissociation and reformation. Consequently, it may not fully capture the exchange kinetics associated with different hydration environments, including those discussed in relation to intermediate water. In contrast, the rk_f_/rk_b_ ratio characterizes the relative balance between the characteristic times of HB dissociation and reformation, thereby providing complementary information on HB exchange dynamics that cannot be obtained from the integrated correlation time alone. However, owing to the simplifications inherent in the present reductionist framework, the rk_f_/rk_b_ ratio and H_D_ value proposed in this study cannot yet be quantitatively validated against experimental hydration measurements or biological data reported in previous studies. To improve agreement with experimental systems, future model refinements should prioritize thermal annealing, realistic polymer density, stereoregularity, and accurate atomic partial-charge assignments. Furthermore, incorporating experimentally relevant water content will be important for reproducing hydration properties more accurately. The kinetic parameters and the rk_f_/rk_b_ ratio were treated as exploratory, simulation-window-dependent descriptors rather than converged material constants. To assess sampling-duration sensitivity directly, P(MPC) was additionally analyzed using 1, 5, and 10 ns trajectories (Figure S12). The rk_f_/rk_b_ ratio changed from 0.93 at 1 ns to 5.06 and 5.03 at 5 and 10 ns, respectively. Although the last two ratios were similar, the individual rate constants continued to vary substantially, and quantitative convergence was not established. This result limits the interpretation of polymer rankings derived from the 1 ns trajectories. The calculated HB populations may also depend on the sampling conditions and the resulting interfacial configurations. In contrast, HB distance distributions, angle distributions, and RDF primarily characterize the spatial organization and geometry of interfacial water. Although these structural descriptors are also influenced by the molecular characteristics of the polymer, they may be less directly dependent on the temporal resolution of the trajectory than descriptors derived from HB kinetics. Furthermore, the spatially restricted nature of polymer–water interactions at solid–liquid interfaces, compared with more accessible polymer–water interactions in liquid–liquid models, may also contribute to differences in the calculated hydration properties. These considerations highlight the importance of balancing molecular model fidelity and computational efficiency when developing a reductionist MD framework for identifying reliable descriptors of interfacial hydration. Furthermore, the non-reactive fixed-charge force fields employed in this study cannot explicitly describe chemical bond formation or cleavage, including proton-transfer reactions associated with ionization and dissociation processes. Recent advances in reactive neural network potentials developed using active learning and enhanced sampling have demonstrated their applicability to complex chemical reactions, such as the oxidative ageing of asphalt [52]. Such approaches may offer promising opportunities for future investigations of reactive processes at polymer–water interfaces. In particular, their ability to capture dynamic changes in chemical states, including ionization and dissociation processes that were simplified in the present study, may provide valuable insights into experimentally relevant phenomena that are difficult to reproduce using conventional non-reactive MD simulations. However, further validation is required to establish the applicability and accuracy of these approaches for the polymer–water systems investigated in this study. Future research should therefore focus on developing a computationally efficient molecular modeling framework that incorporates these improvements while retaining the practical advantages of candidate descriptors such as rk_f_/rk_b_ and the H_D_ value. These parameters may serve as a practical candidate descriptor for computational screening of hydration-related properties associated with biocompatibility, without requiring direct estimation of the absolute amounts of experimentally defined bound or intermediate water. Furthermore, the present analysis suggests that the molecular factors governing interfacial hydration may differ among betaine, nonionic, and amino-acid-based polymers. Therefore, the rk_f_/rk_b_-based approach may provide a simple and mechanistically informative descriptor of HB exchange dynamics without relying solely on the quantification of experimentally defined hydration states. These findings indicate that the rk_f_/rk_b_ ratio captures a distinct dynamic aspect of interfacial hydration—the relative balance between HB dissociation and reformation—that is not represented by τ_HB_ alone.

## 4. Conclusions

The main findings of this study can be summarized as follows:

Using a reductionist solid–liquid interface molecular dynamics (MD) framework, we systematically characterized the interfacial hydration of nonionic, amino-acid-based zwitterionic, betaine-based, and highly polar polymers.

HB distance distributions and radial distribution functions (RDFs) revealed differences in the local organization of water molecules and donor–acceptor configurations at polymer–water interfaces, while HB angle distributions provided complementary information on their geometric characteristics.

HB correlation and kinetic analyses revealed polymer-dependent differences in interfacial hydration dynamics, suggesting that the accessibility and chemical characteristics of polymer side chains, together with differences in the polymer backbone structure, influence the temporal behavior of polymer–water interactions.

The rk_f_/rk_b_ ratio, representing the relative balance between the characteristic timescales of HB dissociation and reformation, was identified as a candidate molecular descriptor of interfacial hydration dynamics. Together with HB populations and integrated correlation times, this ratio provides complementary information that can be obtained using established HB analyses implemented in commonly used MD tools such as GROMACS.

These analyses identify the rk_f_/rk_b_ ratio as a candidate descriptor of interfacial hydration dynamics that may contribute to future AI/ML-assisted molecular screening. However, the ratio exhibited substantial sampling-duration dependence for P(MPC), changing from 0.93 at 1 ns to 5.06 and 5.03 at 5 and 10 ns, respectively (Figure S12). Although the ratios obtained at 5 and 10 ns were similar, the individual rate constants continued to vary, and quantitative convergence was not established. Therefore, the present results do not support reliable quantitative ranking of different polymers based on this descriptor or prediction of their biocompatibility. Further validation using longer and independent simulations, together with experimental hydration and biological data, is required to establish the quantitative reliability and predictive relevance of this candidate descriptor. In addition, the quantitative convergence of the derived kinetic parameters and their relationship with experimental biocompatibility remain to be established. Furthermore, the interfacial area, water-layer thickness, finite-size effects, and surface roughness may influence the spatial organization of interfacial water and the accessibility of polymer functional groups. Since these factors were not systematically evaluated in the present study, their potential effects on the quantitative values and relative trends of the proposed hydration descriptors should be investigated in future studies. Furthermore, the use of UFF for P(NaAC), in contrast to GAFF2 for the other polymer systems, introduces an additional source of uncertainty in the quantitative comparison of interfacial hydration properties. Consequently, differences in the calculated hydration descriptors may reflect not only the intrinsic chemical characteristics of the polymers but also differences in their force-field representations. In addition, the fixed-charge models employed in this study do not explicitly account for environment-dependent electronic polarization, which may affect the predicted polymer–water interactions and hydrogen-bond dynamics. Therefore, systematic evaluation of force-field dependence, including alternative parameterization and partial-charge assignment methods, remains an important subject for future investigation.

Future studies should incorporate key factors identified in this work, including polymer stereoregularity, ionization states, and accurate atomic charge assignments, and develop a more realistic single-interface model of solid–liquid biomaterial interfaces. Comparing RESP and AM1-BCC charge assignments will help clarify the role of atomic charge distributions in interfacial hydration. Furthermore, extending simulation times and comparing the resulting descriptors with experimental hydration and biological data will be essential for evaluating their quantitative reliability and potential utility in biocompatibility-related molecular screening.

## Figures and Tables

**Table 1:** Integrated peak areas of the hydrogen-bond angle distributions shown in Figure 3.

Angle	Water	P(MEA)	P(AC)	P(SPB)	P(CMB)	P(MPC)	P(N^α^-LysAA)	P(N^ε^-LysAA)	P(N^α^-LysMA)	P(NaAC)
0.5–35.5	18.61	36.23	25.84	41.82	38.22	40.44	25.02	26.02	24.12	17.00
36.5–89.5	23.11	20.20	26.42	13.65	15.07	14.82	25.09	22.01	28.37	24.89
90.5–130.5	45.96	38.60	35.93	41.12	42.64	41.38	40.04	42.53	37.35	43.01
131.5–179.5	12.27	4.91	11.77	3.29	3.95	3.21	9.79	9.38	10.11	15.04

**Table 2:** Hydrogen-bond analysis of each polymer at the polymer–water interface (~0.35 nm).

Molecule	HB (*N*/nm^2^)	Pairs (*N*/nm^2^)	Degree of HB Formation (%)	HB/Pairs	N^corr^
P(AC)	23.51	45.16	34.24	0.521	2.3
P(MEA)	8.42	4.20	66.73	2.006	N.D.
P(SPB)	20.26	6.47	75.80	3.133	1.2
P(CMB)	18.95	6.79	73.61	2.790	−0.3
P(MPC)	18.11	5.35	77.19	3.384	−0.7
P(N^α^-LysAA)	26.60	29.59	47.33	0.899	N.D.
P(N^ε^-LysAA)	21.73	21.69	50.05	1.002	N.D.
P(N^α^-LysMA)	15.48	18.62	45.39	0.831	N.D.
P(NaAC)	44.85	98.03	31.39	0.458	5.9

Cutoff criteria: Donor–acceptor distance, 0.35 nm; deviation angle, 30°. HB: Number of D–H···O–A triplets satisfying both distance and angular criteria at the interface. Pairs: Number of non-HB donor–acceptor configurations that satisfy the distance criterion but do not satisfy the angular HB criterion. In the GROMACS output used here, HB and Pairs are mutually exclusive categories. Degree of HB formation (%) = HB/(HB + Pairs) × 100. HB/Pairs: Indicator of preferential water orientation arising from HB formation. N^corr^: Defective index of intermolecular HB among water molecules, derived from polarization analysis of Raman spectra reported by Kitano et al. A larger positive value indicates greater disruption of the HB network.

**Table 3:** Hydrogen-bond analysis of each polymer at the polymer–water interface (~1.0 nm).

Molecule	HB (*N*/nm^2^)	Pairs (*N*/nm^2^)	Degree of HB Formation (%)	HB/Pairs	N^corr^
P(AC)	310.56	2285.68	11.96	0.136	2.3
P(MEA)	146.30	905.00	13.92	0.162	N.D.
P(SPB)	218.11	1366.00	13.77	0.160	1.2
P(CMB)	211.11	1276.14	14.19	0.165	−0.3
P(MPC)	245.59	1330.13	15.59	0.185	−0.7
P(N^α^-LysAA)	320.39	1873.49	14.60	0.171	N.D.
P(N^ε^-LysAA)	275.13	1623.54	14.49	0.169	N.D.
P(N^α^-LysMA)	226.06	1309.86	14.72	0.173	N.D.
P(NaAC)	643.24	3870.09	14.25	0.166	5.9

Cutoff criteria: Donor–acceptor distance, 1.0 nm; angular deviation, 30°. The definitions of HB, Pairs, Degree of HB formation, and HB/Pairs follow Table 2, with the extended distance criterion. Notes: The resulting counts are reported as extended-cutoff geometric contact measures and are not interpreted as conventional HBs at donor–acceptor separations beyond 0.35 nm.

**Table 4:** HB dissociation and reformation rate constants (k_f_ and k_b_), reciprocal characteristic times (rk_f_ and rk_b_) and their ratio, and τ_HB_ for polymer–water interface.

Molecule	Lifetime					
	k_f_ (ps^−1^)	k_b_ (ps^−1^)	rk_f_ (ps)	rk_b_ (ps)	rk_f_/rk_b_	τ_HB_ (ps)
Bulk water	0.590	1.519	1.70	0.66	2.58	5.94
P(AC)	0.063	0.079	15.82	12.60	1.26	37.10
P(MEA)	0.099	0.301	10.07	3.32	3.03	33.57
P(SPB)	0.024	0.042	42.24	24.07	1.76	63.41
P(CMB)	0.031	0.056	32.25	17.71	1.82	56.88
P(MPC)	0.016	0.015	62.86	67.79	0.93	75.82
P(N^α^-LysAA)	0.027	0.019	36.97	53.36	0.69	54.52
P(N^ε^-LysAA)	0.034	0.046	29.82	21.73	1.37	52.20
P(N^α^-LysMA)	0.099	0.216	10.14	4.64	2.19	35.53
P(NaAC)	0.015	0.004	65.69	275.11	0.24	74.36

**Table 5:** HB dissociation and reformation rate constants, reciprocal characteristic times and relative hydration-disruption indices calculated from water–water HB kinetics in polymer-containing systems.

Molecule	Lifetime					H_D_ Value	N^corr^
	k_f_ (ps^−1^)	k_b_ (ps^−1^)	rk_f_ (ps)	rk_b_ (ps)	rk_f_/rk_b_		
Bulk water	0.590	1.519	1.70	0.66	2.58	1.00	N.A.
P(AC)	0.565	1.457	1.77	0.69	2.58	1.00	2.3
P(MEA)	0.607	1.847	1.65	0.54	3.05	1.18	N.A.
P(SPB)	0.529	1.789	1.89	0.56	3.38	1.31	1.2
P(CMB)	0.541	1.654	1.85	0.60	3.06	1.19	−0.3
P(MPC)	0.507	1.716	1.97	0.58	3.38	1.31	−0.7
P(N^α^-LysAA)	0.502	1.329	1.99	0.75	2.65	1.03	N.A.
P(N^ε^-LysAA)	0.562	1.558	1.78	0.64	2.77	1.08	N.A.
P(N^α^-LysMA)	0.595	1.889	1.68	0.53	3.18	1.23	N.A.
P(NaAC)	0.333	0.537	3.01	1.86	1.61	0.63	5.9

H_D_ value: **T**he ratio of rk_f_/rk_b_ calculated for water–water HBs in each polymer-containing system to the corresponding ratio for water–water HBs in the reference bulk-water system. N^corr^: Defective index of intermolecular HB among water molecules, derived from polarization analysis of Raman spectra reported by Kitano et al. A larger positive value indicates greater disruption of the HB network.

## Data Availability

The data supporting this article, including raw data for Figure S1–12, and the MD simulation conditions and raw data, are available in the Zenodo repository at 10.5281/zenodo.23527762.

## Supplementary Materials

The following supporting information can be downloaded at https://www.mdpi.com/article/10.3390/polym18212677/s1: Figure S1: Simulation cell size, density, and simulation conditions after density equilibration; Figure S2: Interfacial models of each polymer after density equilibration; Figure S3: Interfacial models of each polymer after density equilibration; Figure S4: Density profile of each polymer; Figure S5: Block-averaged number of HBs during the NPT production simulation; Figure S6: Statistical analysis of hydrogen-bond (HB) populations over the entire 0–1000 ps NPT production trajectory; Figure S7: Detailed analysis of hydrogen bond amount under cutoff conditions of 0.35–1.0 nm and 30°; Figure S8: Detailed analysis of hydrogen bond amount under cutoff conditions of 0.35–1.0 nm and 30°; Figure S9: Intermittent hydrogen-bond autocorrelation analysis of polymer–water interactions; Figure S10: HB–non-HB contact cross-correlation analysis of polymer–water interactions; Figure S11: HB dissociation and reformation rate constants (k_f_ and k_b_), reciprocal characteristic times (rk_f_ and rk_b_) and their ratio, and τ_HB_ for polymer–water interface; Figure S12: Comparison of hydrogen-bond dissociation and reformation kinetic parameters for P(MPC) obtained from 1, 5 and 10 ns production MD simulations.
