# Supplementary material for: A Reductionist Molecular Dynamics Framework for Identifying Molecular Descriptors of Hydration at Solid–Liquid Biomaterial Interfaces
Source: Polymers (Basel). 2026 Oct 28;18(21):2677. doi: 10.3390/polym18212677 (PMC13765684; doi:10.3390/polym18212677)
Supplement: Supplementary file 1 [file polymers-18-02677-s001.zip › polymers-4639976-supplementary.pdf]

A Reductionist Molecular Dynamics Framework for Identifying Molecular Descriptors of Hydration at Solid–Liquid Biomaterial Interfaces

Tomoya Nakago [1], Kohei Shiraishi [1]

Major in Systems Engineering, Graduate School of Systems Engineering, Kindai University, 1 Umenobe, Takaya, Higashi-hiroshima, Hiroshima 739-2116, Japan.

Correspondence: Kohei Shiraishi(siraisi@hiro.kindai.ac.jp)

Supplemental Information

Figure S-1. Simulation cell size, density, and simulation conditions after density equilibration.

Molecule	Temperature (K)	Pressure (atm)	Cell size (nm)			Density (g/cm^3)
			x	y	z	
Bulk water	310.15	1.0	4.49	4.49	4.49	0.99
P(AC)	310.15	1.0	3.79	3.79	8.19	1.07
P(MEA)	310.15	1.0	4.11	4.11	8.81	1.04
P(SPB)	310.15	1.0	4.81	4.81	10.23	1.00
P(CMB)	310.15	1.0	4.32	4.32	9.23	1.14
P(MPC)	310.15	1.0	4.71	4.71	10.03	1.07
P(N$^{\alpha}$-LysAA)	310.15	1.0	4.28	4.28	9.15	1.13
P(N$^{\varepsilon}$-LysAA)	310.15	1.0	4.30	4.30	9.18	1.12
P(N$^{\alpha}$-LysMA)	310.15	1.0	4.43	4.43	9.45	1.10
P(NaAC)	310.15	1.0	3.52	3.52	9.25	1.20

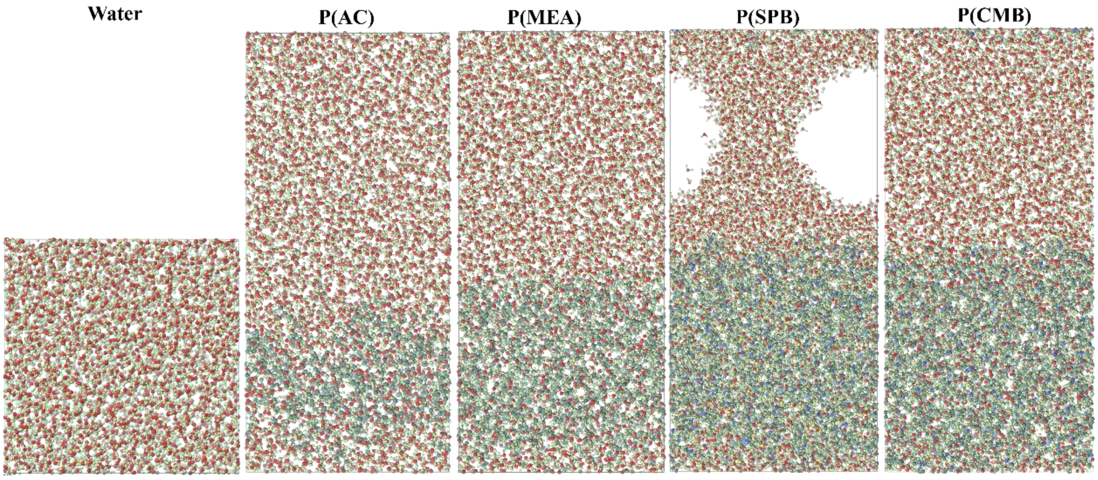

Figure S-2. Interfacial models of each polymer after density equilibration. Note: Atoms are color-coded as follows: hydrogen (white), oxygen (red), nitrogen (blue), carbon (gray).

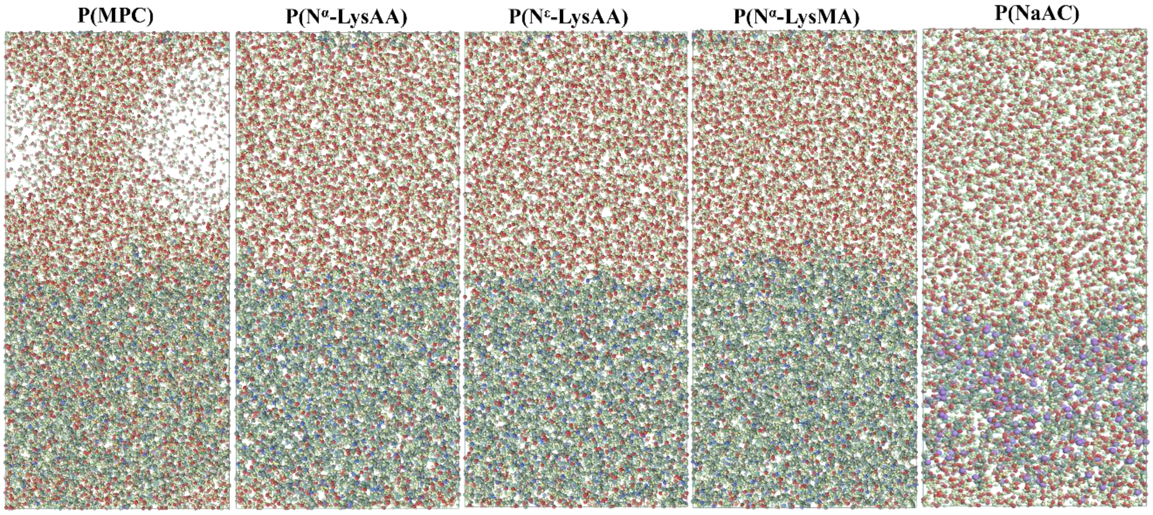

Figure S-3. Interfacial models of each polymer after density equilibration. Note: Atoms are color-coded as follows: hydrogen (white), oxygen (red), nitrogen (blue), carbon (gray), phosphorus (orange), and sodium (purple).

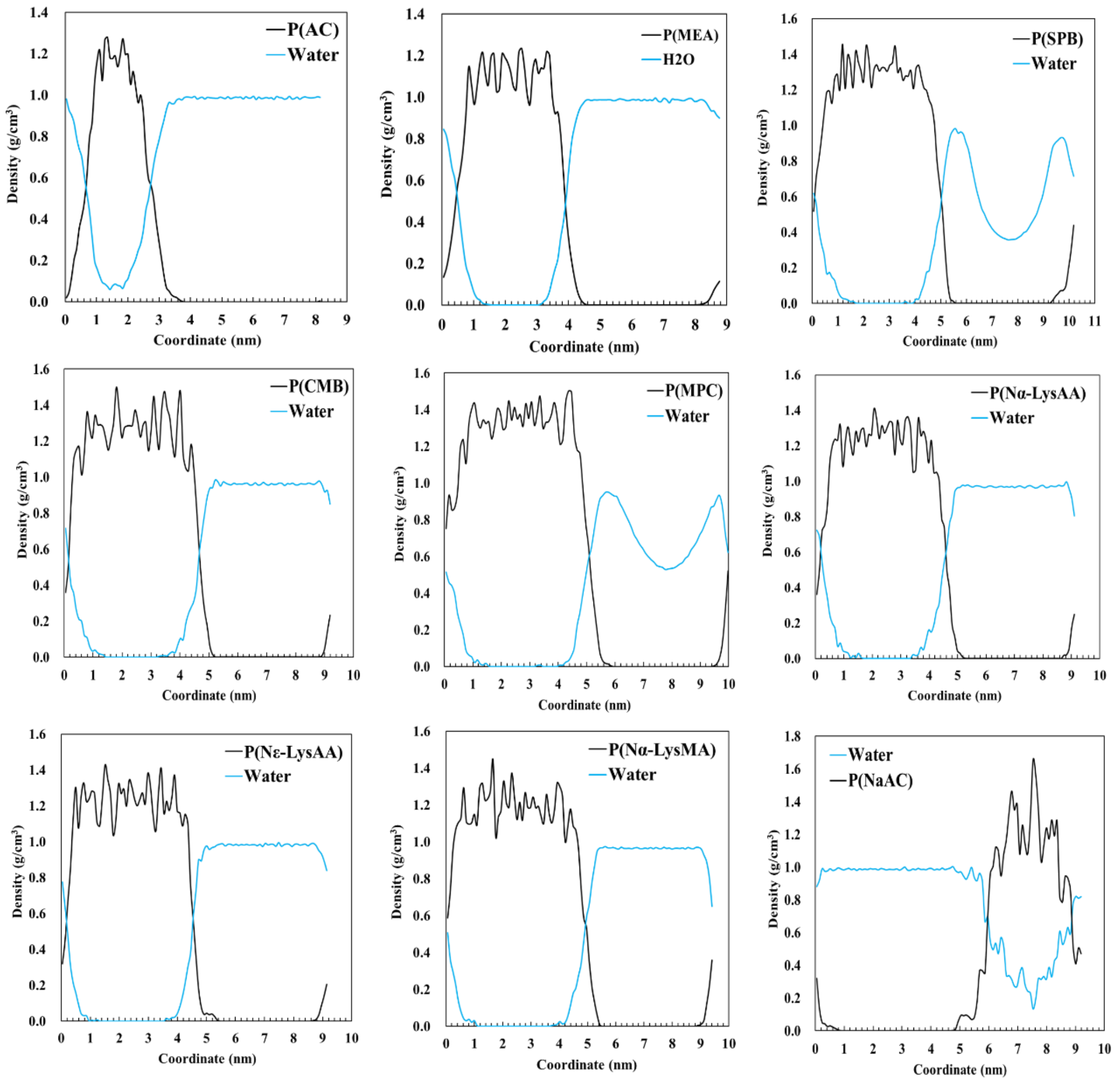

Figure S-4. Density profile of each polymer.

Figure S-5. Block-averaged number of HBs during the NPT production simulation.

Molecule	Time Interval (ps) 0–200	200–400	400–600	600–800	800–1000	Overall Mean	SD	SE	CV (%)
	Block-Averaged Number of HBs								
P(AC)	334.82	338.35	338.03	337.25	344.16	338.52	3.44	1.54	1.02
P(MEA)	143.13	143.85	144.99	141.98	137.05	142.20	3.08	1.38	2.17
P(SPB)	452.68	460.20	467.64	475.65	485.25	468.28	12.76	5.71	2.73
P(CMB)	335.12	346.91	356.71	363.37	368.66	354.15	13.39	5.99	3.78
P(MPC)	387.64	398.30	402.33	408.24	411.10	401.52	9.23	4.13	2.30
P(N^{α}-LysAA)	479.90	484.31	490.61	489.92	493.43	487.63	5.45	2.44	1.12
P(N^{ε}-LysAA)	390.16	395.19	407.34	407.34	410.90	402.19	8.98	4.02	2.23
P(N^{α}-LysMA)	298.81	297.20	304.94	310.33	314.07	305.07	7.24	3.24	2.37
P(NaAC)	538.52	550.27	555.48	554.17	563.83	552.45	9.22	4.13	1.67

Overall Mean: Mean HB number calculated over the entire 0–1000 ps trajectory. **Standard deviation (SD)**: Standard deviation of the five block-averaged HB numbers. **Standard error (SE)**: Standard error calculated from the five block-averaged HB numbers. **Coefficient of variation (CV)**: Ratio of the SD of the block-averaged HB numbers to the overall mean, expressed as a percentage.

Figure S-6. Statistical analysis of hydrogen-bond (HB) populations over the entire 0–1000 ps NPT production trajectory.

Molecule	Mean	SD	CV (%)
P(AC)	338.51	9.78	2.89
P(MEA)	142.19	6.73	4.74
P(SPB)	468.32	13.58	2.90
P(CMB)	354.17	14.08	3.97
P(MPC)	401.54	10.47	2.61
P(N^{α}-LysAA)	487.62	10.04	2.06
P(N^{ε}-LysAA)	401.32	10.87	2.71
P(N^{α}-LysMA)	304.07	12.69	4.17
P(NaAC)	552.48	11.67	2.11

SD: Standard deviation of the HB numbers calculated from all sampled frames over the 0–1000 ps trajectory. **CV**: Ratio of the SD of the HB numbers to the overall mean HB number, expressed as a percentage.

Figure S-7. Detailed analysis of hydrogen bond amount under cutoff conditions of 0.35–1.0 nm and 30°.

Molecule	Charge	HB (N/nm^2)			Pairs (N/nm^2)			HB/Pairs		
		0.35	0.70	1.00	0.35	0.70	1.00	0.35	0.70	1.00
P(AC)	Total	23.51	97.95	310.56	45.16	632.67	2285.68	0.521	0.155	0.136
	Anion	23.51	97.95	310.56	45.16	632.67	2285.68	0.521	0.155	0.136
	Cation	N.D.	N.D.	N.D.	N.D.	N.D.	N.D.	N.D.	N.D.	N.D.
P(MEA)	Total	8.42	37.50	146.30	4.20	236.02	905.00	2.006	0.159	0.162
	Anion	8.42	37.50	146.30	4.20	236.02	905.00	2.006	0.159	0.162
	Cation	N.D.	N.D.	N.D.	N.D.	N.D.	N.D.	N.D.	N.D.	N.D.
P(SPB)	Total	20.26	76.31	218.11	6.47	375.66	1366.00	3.133	0.203	0.160
	Anion	19.24	64.22	153.60	4.11	224.29	820.25	4.681	0.286	0.187
	Cation	1.01	12.09	64.50	2.36	151.36	545.75	0.431	0.080	0.118
P(CMB)	Total	18.95	71.21	211.11	6.79	361.71	1276.14	2.790	0.197	0.165
	Anion	18.95	59.74	169.88	6.58	275.50	1002.01	2.880	0.217	0.170
	Cation	N.D.	11.46	41.23	N.D.	86.22	274.13	N.D.	0.133	0.150
P(MPC)	Total	18.11	83.37	245.59	5.35	364.74	1330.13	3.384	0.229	0.185
	Anion	18.11	78.28	212.10	5.12	285.73	1096.55	3.536	0.274	0.193
	Cation	N.D.	5.08	33.49	N.D.	79.01	233.58	N.D.	0.064	0.143
P(N^{α}-LysAA)	Total	26.60	112.19	320.39	29.59	517.86	1873.49	0.899	0.217	0.171
	Anion	20.22	61.19	152.63	6.59	212.18	794.41	3.070	0.288	0.192
	Cation	6.37	50.99	167.76	23.01	305.68	1079.08	0.277	0.167	0.155
P(N^{ε}-LysAA)	Total	21.73	87.77	275.13	21.69	440.95	1623.54	1.002	0.199	0.169
	Anion	14.70	44.40	125.09	6.45	193.50	716.04	2.279	0.229	0.175
	Cation	7.03	43.37	150.04	15.24	247.45	907.51	0.461	0.175	0.165
P(N^{α}-LysMA)	Total	15.48	73.72	226.06	18.62	344.48	1309.86	0.831	0.214	0.173
	Anion	9.81	35.75	99.93	5.04	140.82	555.05	1.946	0.254	0.180
	Cation	5.67	37.97	126.14	13.58	203.66	754.81	0.417	0.186	0.167
P(NaAC)	Total	44.85	216.86	643.24	98.03	1164.28	3870.09	0.458	0.186	0.166
	Anion	44.61	158.78	446.24	40.65	763.08	2549.05	1.097	0.208	0.175
	Cation	0.25	58.08	197.00	57.38	401.20	1321.04	0.004	0.145	0.149

Cutoff criteria: Donor–acceptor distance, 0.35, 0.70, 1.00 nm; deviation angle, 30°. **HB**: Number of D–H···O–A triplets satisfying both distance and angular criteria at the interface. **Pairs**: Number of non-HB donor–acceptor configurations that satisfy the distance criterion but do not satisfy the angular HB criterion. In the GROMACS output used here, HB and Pairs are mutually exclusive categories. **HB/Pairs**: Indicator of preferential water orientation

arising from HB formation.

Figure S-8. Detailed analysis of hydrogen bond amount under cutoff conditions of 0.35–1.0 nm and 30°.

Molecule	Charge	HB Contribution (%)			Degree of HB formation (%)		
		0.35	0.70	1.00	0.35	0.70	1.00
P(AC)	Total	N.A.	N.A.	N.A.	34.2	13.4	12.0
	Anion	100.0	100.0	100.0	34.2	13.4	12.0
	Cation	N.A.	N.A.	N.A.	N.A	N.A	N.A
P(MEA)	Total	N.A.	N.A.	N.A.	66.7	13.7	13.9
	Anion	100.0	100.0	100.0	66.7	13.7	13.9
	Cation	N.A.	N.A.	N.A.	N.A	N.A	N.A
P(SPB)	Total	N.A.	N.A.	N.A.	75.8	16.9	13.8
	Anion	95.0	84.2	70.4	82.4	22.3	15.8
	Cation	5.0	15.8	29.6	30.1	7.4	10.6
P(CMB)	Total	N.A.	N.A.	N.A.	73.6	16.4	14.2
	Anion	100.0	83.9	80.5	74.2	17.8	14.5
	Cation	N.A.	16.1	19.5	N.A.	11.7	13.1
P(MPC)	Total	N.A.	N.A.	N.A.	77.2	18.6	15.6
	Anion	100.0	93.9	86.4	78.0	21.5	16.2
	Cation	N.A.	6.1	13.6	N.A.	6.0	12.5
P(N^{α}-LysAA)	Total	N.A.	N.A.	N.A.	47.3	17.8	14.6
	Anion	76.0	54.5	47.6	75.4	22.4	16.1
	Cation	24.0	45.5	52.4	21.7	14.3	13.5
P(N^{ε}-LysAA)	Total	N.A.	N.A.	N.A.	50.0	16.6	14.5
	Anion	67.7	50.6	45.5	69.5	18.7	14.9
	Cation	32.3	49.4	54.5	31.6	14.9	14.2
P(N^{α}-LysMA)	Total	N.A.	N.A.	N.A.	45.4	17.6	14.7
	Anion	63.4	48.5	44.2	66.1	20.2	15.3
	Cation	36.6	51.5	55.8	29.4	15.7	14.3
P(NaAC)	Total	N.A.	N.A.	N.A.	31.4	15.7	14.3
	Anion	99.5	73.2	69.4	52.3	17.2	14.9
	Cation	0.6	26.8	30.6	0.4	12.6	13.0

HB contribution ratio (%): An indicator of the relative contribution of each functional group to hydrogen bond formation under the specified conditions. **Degree of HB formation (%)** =

HB/(HB + Pairs) × 100.

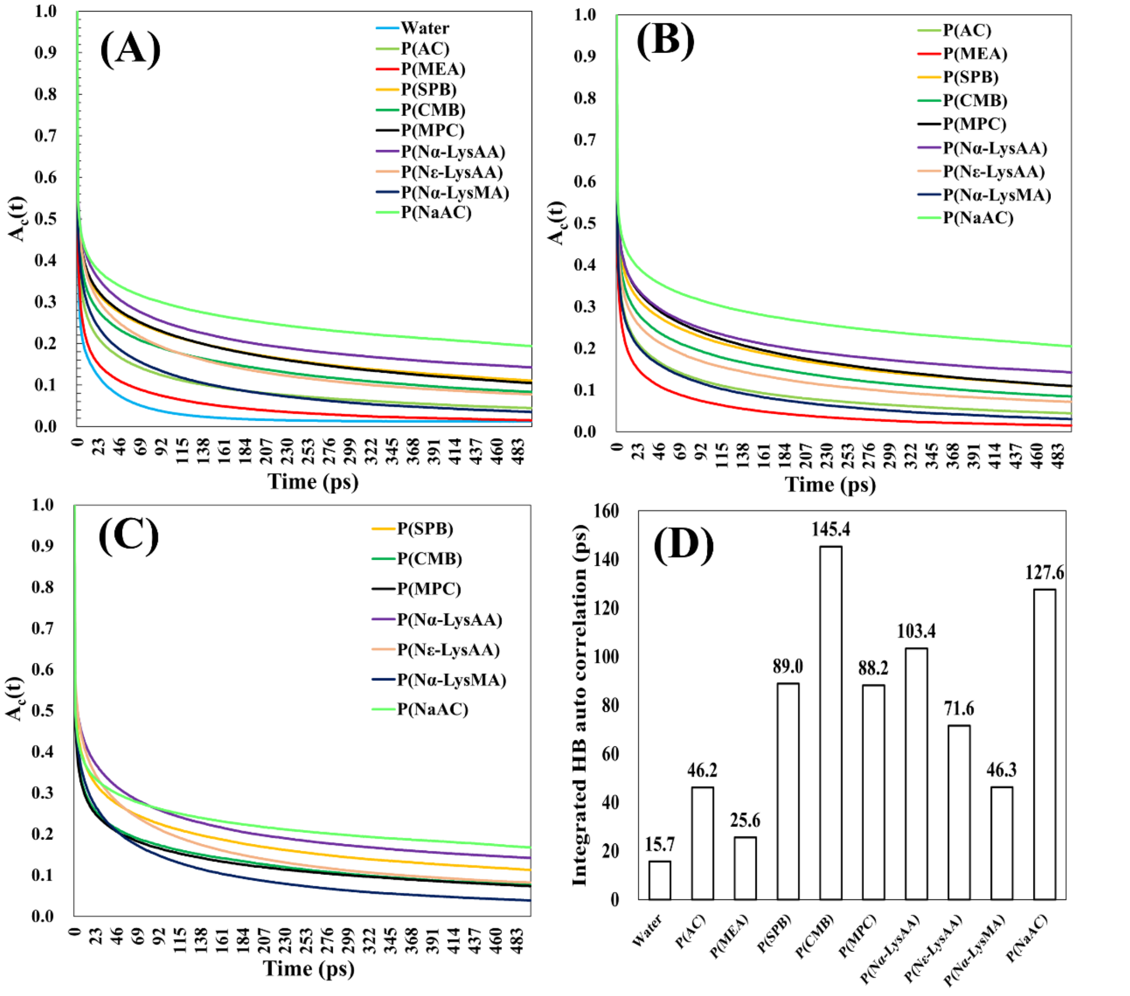

Figure S-9. Intermittent hydrogen-bond autocorrelation analysis of polymer–water interactions. Cutoff conditions: Donor–acceptor distance = 1.00 nm; angular deviation for HB formation = 30°. (A) Intermittent HB autocorrelation functions for the entire polymer system. (B) Intermittent HB autocorrelation functions associated with the anionic components. (C) Intermittent HB autocorrelation functions associated with the cationic components. Systems exhibiting no detectable correlations under these conditions were excluded from the analysis. (D) Finite-time integrated values of the intermittent HB autocorrelation functions shown in panel (A), calculated using the trapezoidal rule, as employed in the angular distribution analysis (Section 2.2.3).

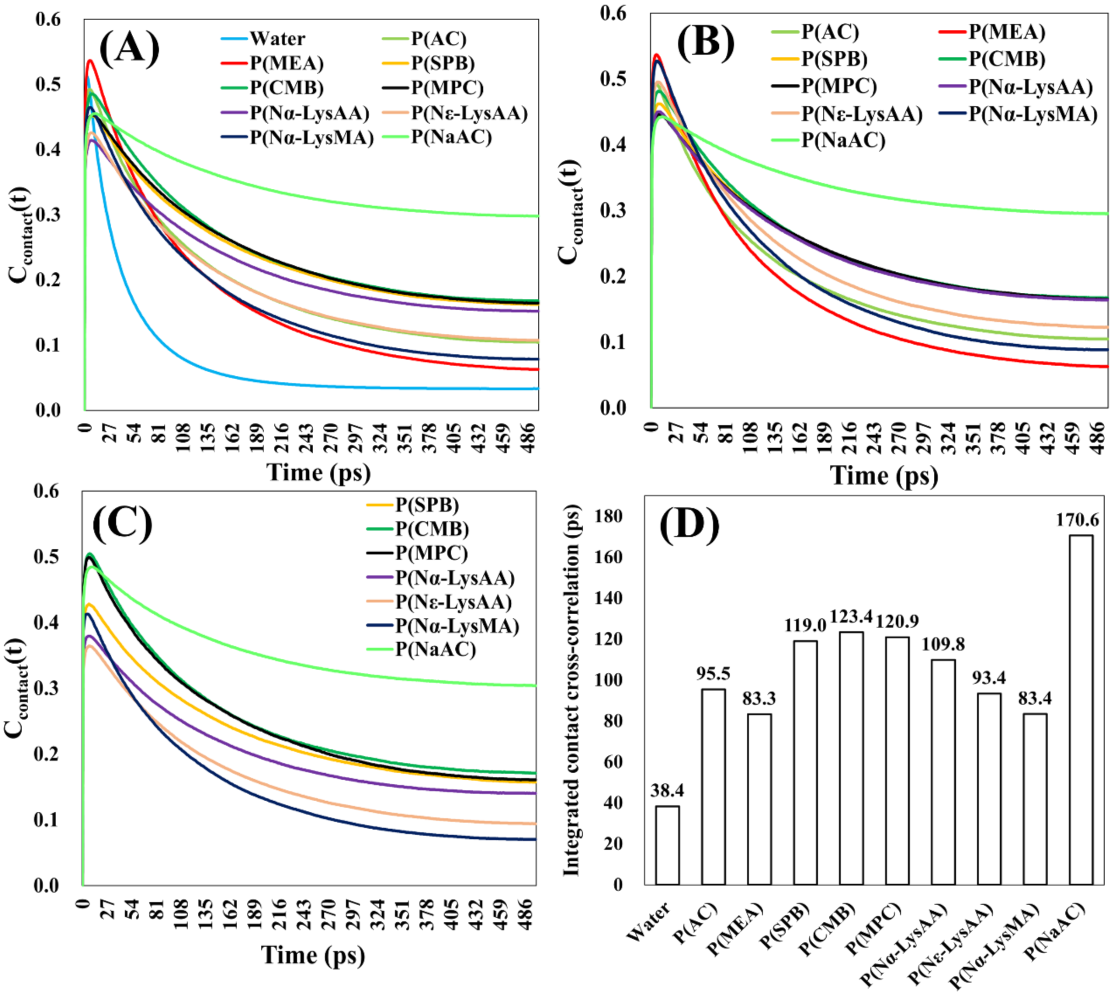

Figure S-10. HB–non-HB contact cross-correlation analysis of polymer–water interactions. Cutoff conditions: Donor–acceptor distance = 1.00 nm; angular deviation for HB formation = 30°. (A) Contact cross-correlation functions for the entire polymer system. (B) Contact cross-correlation functions associated with the anionic components. (C) Contact cross-correlation functions associated with the cationic components. Systems exhibiting no detectable correlations under these conditions were excluded from the analysis. (D) Finite-time integrals of the contact cross-correlation functions shown in panel (A), calculated using the trapezoidal rule, as employed in the angular distribution analysis (Section 2.2.3).

Figure S-11. HB dissociation and reformation rate constants (k_f and k_b), reciprocal characteristic times (rk_f and rk_b) and their ratio, and τ_{HB} for polymer–water interface.

Molecule	Lifetime					
	k_f (ps^{-1})	k_b (ps^{-1})	rk_f (ps)	rk_b (ps)	rk_f/rk_b	τ_{HB} (ps)
Water	0.673	0.200	1.49	5.00	0.30	9.59
P(AC)	0.184	0.038	5.44	26.23	0.21	19.85
P(MEA)	0.321	0.064	3.12	15.63	0.20	15.38
P(SPB)	0.091	0.018	11.03	54.56	0.20	28.75
P(CMB)	0.110	0.020	9.06	49.28	0.18	25.87
P(MPC)	0.083	0.017	11.99	58.37	0.21	30.23
P(N^{α}-LysAA)	0.081	0.017	12.28	57.64	0.21	29.11
P(N^{ε}-LysAA)	0.103	0.028	9.75	35.09	0.28	28.56
P(N^{α}-LysMA)	0.161	0.046	6.20	21.51	0.29	23.99
P(NaAC)	0.073	0.008	13.77	126.88	0.11	27.70

The dissociation and reformation rate constants (k_f and k_b[ps^{-1}]) were estimated using the Luzar–Chandler kinetic analysis. Their reciprocal characteristic times are defined as $rk_f = 1/k_f$ and $rk_b = 1/k_b$ (ps), and the dimensionless ratio rk_f/rk_b was evaluated.

Figure S-12. Comparison of hydrogen-bond dissociation and reformation kinetic parameters for P(MPC) obtained from 1, 5 and 10 ns production MD simulations.

Simulation time (ns)	k_f (ps^{-1})	k_b (ps^{-1})	rk_f (ps)	rk_b (ps)	rk_f/rk_b
1	0.016	0.015	62.86	67.79	0.93
5	0.012	0.060	84.90	16.77	5.06
10	0.007	0.037	135.22	26.91	5.03

Note: k_f and k_b rate constants describe HB dissociation and reformation, respectively. The corresponding characteristic timescales were calculated as $rk_f = 1/k_f$ and $rk_b = 1/k_b$. The dimensionless ratio (rk_f/rk_b) represents the relative characteristic timescales of HB dissociation and reformation. The incomplete decay of the HB autocorrelation functions indicates that the kinetic parameters remain sensitive to simulation duration and have not been demonstrated to be quantitatively converged.
